# MIRO2-mediated mitochondrial transfer from cancer cells induces cancer-associated fibroblast differentiation

**DOI:** 10.1038/s43018-025-01038-6

**Published:** 2025-08-28

**Authors:** Michael Cangkrama, Huan Liu, Xiaoyu Wu, Josephine Yates, James Whipman, Christoph G. Gäbelein, Mai Matsushita, Luca Ferrarese, Sibilla Sander, Francesc Castro-Giner, Simran Asawa, Magdalena K. Sznurkowska, Manfred Kopf, Jörn Dengjel, Valentina Boeva, Nicola Aceto, Julia A. Vorholt, Sabine Werner

**Affiliations:** 1 Institute of Molecular Health Sciences, Department of Biology, ETH Zurich, Zurich, Switzerland; 2 Department of Computer Science, ETH Zurich, Zurich, Switzerland; 3 Institute of Microbiology, Department of Biology, ETH Zurich, Zurich, Switzerland; 4 Department of Biology, University of Fribourg, Fribourg, Switzerland; 5 Swiss Institute for Bioinformatics (SIB), Lausanne, Switzerland; 6 UMR-S1016 Institut Cochin, Université de Paris, Paris, France; 7 Present Address: Whitehead Institute for Biomedical Research, Cambridge, MA USA

**Keywords:** Cancer microenvironment, Organelles, Cancer

## Abstract

Cancer-associated fibroblasts (CAFs) are key components of the tumor microenvironment that commonly support cancer development and progression. Here we show that different cancer cells transfer mitochondria to fibroblasts in cocultures and xenograft tumors, thereby inducing protumorigenic CAF features. Transplantation of functional mitochondria from cancer cells induces metabolic alterations in fibroblasts, expression of CAF markers and release of a protumorigenic secretome and matrisome. These features promote tumor formation in preclinical mouse models. Mechanistically, the mitochondrial transfer requires the mitochondrial trafficking protein MIRO2. Its depletion in cancer cells suppresses mitochondrial transfer and inhibits CAF differentiation and tumor growth. The clinical relevance of these findings is reflected by the overexpression of *MIRO2* in tumor cells at the leading edge of epithelial skin cancers. These results identify mitochondrial transfer from cancer cells to fibroblasts as a driver of tumorigenesis and provide a rationale for targeting MIRO2 and mitochondrial transfer in different malignancies.

## Abstract (web summary)

Cangkrama et al. show that tumor cells from various cancer types use the mitochondrial trafficking protein MIRO2 and nanotubes to transfer mitochondria into surrounding fibroblasts, thereby inducing cancer-associated fibroblast differentiation and subsequent tumor growth.

## Main

Mitochondria are central to energy conversion and signaling events and engage in metabolism and cell fate decisions in health and disease^1,2^. Once an alphaproteobacterial species that evolved into an organelle^3^, mitochondria persist as functionally specialized units, carrying the ability to move between cells. This phenomenon, known as mitochondrial transfer^4,5^, has emerged as a powerful strategy for tissue revitalization and rejuvenation in injured or diseased organs^6^. Recent studies have identified important roles of mitochondrial dynamics in cancer, revealing how mitochondrial transfer contributes to metabolic heterogeneity among tumor cells and influences disease outcomes and treatment responses^7,8^. Mitochondrial transfer can occur through gap junctions, extracellular vesicles, direct mitochondrial release and uptake^9^ or tunneling nanotubes (TNTs), which are thin membranous structures that form dynamic connections between cells^9^. Transfer of mitochondria from stromal or immune cells in the tumor microenvironment to cancer cells has also been reported, which promoted tumor growth. For example, mitochondrial transfer from CD8^+^ T cells, mesenchymal stem cells or cancer-associated fibroblasts (CAFs) into cancer cells has been described for different tumors, resulting in enhanced cancer cell proliferation, motility and lactate metabolism^10–13^. However, the opposite process—mitochondrial transfer from cancer cells to stromal cells, including fibroblasts—has not been reported, although this may have important consequences for the fibroblast phenotype. Here, we identify mitochondrial transfer from cancer cells to fibroblasts as a key regulator of CAF differentiation.

## Results

### Cancer cells transfer mitochondria to fibroblasts through TNTs

**Fig. 1:**
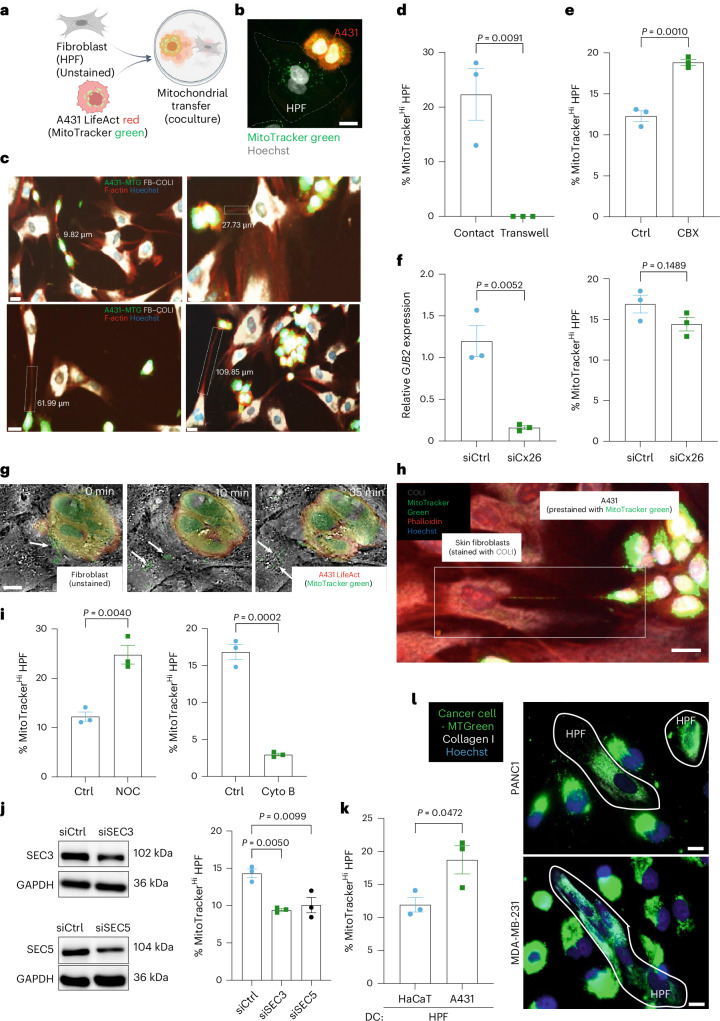
Cancer cells transfer mitochondria to fibroblasts through TNTs. **a**, Coculture setup with LifeAct A431 cells (red) stained with MitoTracker green and unstained HPFs. This image was created with BioRender.com. **b**, Immunofluorescence images of the cocultures, counterstained with Hoechst. **c**, Representative photomicrographs of cocultures of A431 cells prestained with MitoTracker green and HPFs immunostained for COLI (white) and counterstained with phalloidin (red) and Hoechst (blue). TNT-like structures are indicated by white rectangles together with their length (*n* = 3 A431–HPF cocultures). **d**, Percentage of MitoTracker-high HPFs in direct or transwell coculture with A431 cells (*n* = 3 cocultures per setup). **e**, Percentage of MitoTracker-high HPFs after coculture with A431 cells in the presence of carbenoxolone (CBX) or vehicle (*n* = 3 cocultures per treatment group). **f**, RT–qPCR for *GJB2* (encoding connexin 26) relative to *RPL27* using RNA from A431 cells transfected with control (scrambled) or connexin 26 (Cx26) siRNA, and percentage of MitoTracker-high HPFs after coculture of siCtrl or siCx26 A431 cells (*n* = 3 cultures per group). **g**, Holotomographic imaging showing mitochondrial transfer (white arrows) from A431 LifeAct–MitoTracker green cells to HPFs (unstained) (Supplementary Video 1). **h**, Representative image of a coculture of A431 cells stained with MitoTracker green and HPFs, immunostained for COLI and counterstained with phalloidin and Hoechst (*n* = 3 A431–HPF cocultures). White arrows point to TNT-like structures. **i**, Percentage of MitoTracker-high HPFs after coculture with A431 cells in the presence of nocodazole (Noc), dihydrocytochalasin B (Cyto B) or vehicle (*n* = 3 cocultures per treatment group). **j**, Western blot analysis for SEC3 and SEC5 using lysates from A431 cells transfected with siCtrl, siSEC3 or siSEC5 (*n* = 2 cultures per group). Graph shows the percentage of MitoTracker-high HPFs after coculture with MitoTracker-stained control or SEC3–SEC5-knockdown A431 cells (*n* = 3 cocultures per group). **k**, Percentage of MitoTracker-high HPFs after coculture with HaCaT or A431 cells (*n* = 3 cocultures per cell line). **l**, Representative immunofluorescence images depicting cocultures of MDA-MB-231 and PANC1 cells prestained with MitoTracker green and HPFs immunostained for COLI (white) and counterstained with Hoechst (blue) (*n* = 3 cocultures per cell line). Graphs show the mean ± s.e.m. An unpaired two-sided Student’s *t*-test (**d**–**f**,**i**,**k**) or two-sided one-way ANOVA with Bonferroni post hoc multiple comparison test (**j**) was used to determine statistical significance. Scale bars, 50 μm (**b**,**g**), 20 μm (**c**) or 25 μm (**h**,**l**). Source data

Given the association of the CAF phenotype with metabolic alterations^14,15^, we tested whether cancer cells transfer their mitochondria to fibroblasts using cocultures of early-passage human primary skin fibroblasts (HPFs) with highly malignant A431 vulvar carcinoma cells^16^. A431 cells stably expressing fluorescently labeled actin (LifeAct A431) were incubated with MitoTracker green, which stains mitochondria in living cells (Fig. 1a). This approach was chosen because of the strong fluorescence signal of MitoTracker green. Only some HPFs in close proximity to A431 cells became positive for MitoTracker green after a 24-h coculture, indicating that they received cancer cell mitochondria (Fig. 1b). They were clearly discernible against the weak background fluorescence, which may have resulted from dye leakage—a previously reported limitation of MitoTracker dyes that often produces false-positive results^17^.

We observed elongated, thin bridges with a length of 10–100μm between cancer cells and HPFs (Fig. 1c), suggesting that the transfer of mitochondria occurs through TNTs. Consistently, we did not observe MitoTracker-high HPFs in a transwell assay, which does not allow communication through TNTs and gap junctions^18^ (Fig. 1d). Treatment of the cocultures with the gap junction inhibitor carbenoxolone even increased the transfer, as shown by flow cytometry analysis of MitoTracker-high cells, and knockdown of connexin 26, a major connexin in skin cancer cells^19^, had no effect (Fig. 1e,f). These data further suggest that the transfer occurs through TNTs. This was confirmed by real-time holotomographic imaging (Fig. 1g and Supplementary Video 1). Phalloidin combined with MitoTracker staining revealed actin-containing TNTs transferring mitochondria from A431 cancer cells to HPFs (Fig. 1h). Because TNTs include actin and, in some cases, also microtubules^20^, we explored the requirement of these cytoskeletal components for mitochondrial transfer. Treatment of the cocultures with the microtubule polymerization inhibitor nocodazole even enhanced the transfer, while the actin polymerization inhibitor dihydrocytochalasin B had a strong inhibitory effect (Fig. 1i). Knockdown of the exocyst complex components SEC3 (EXOC1) and SEC5 (EXOC2), which have a documented role in the regulation of the actin cytoskeleton and in TNT formation^10^, also reduced the transfer (Fig. 1j). Mitochondrial transfer was also observed in cocultures of HPFs with immortalized but nontumorigenic human keratinocytes (HaCaT cells^21^). However, their transfer efficiency was significantly lower compared to A431 cancer cells (Fig. 1k). In addition, MDA-MB-231 breast cancer and PANC1 pancreatic cancer cells transferred mitochondria to fibroblasts, demonstrating that this process occurs in different types of cancer cells (Fig. 1l).

### Mitochondrial transfer to fibroblasts occurs in vitro and in vivo

**Fig. 2:**
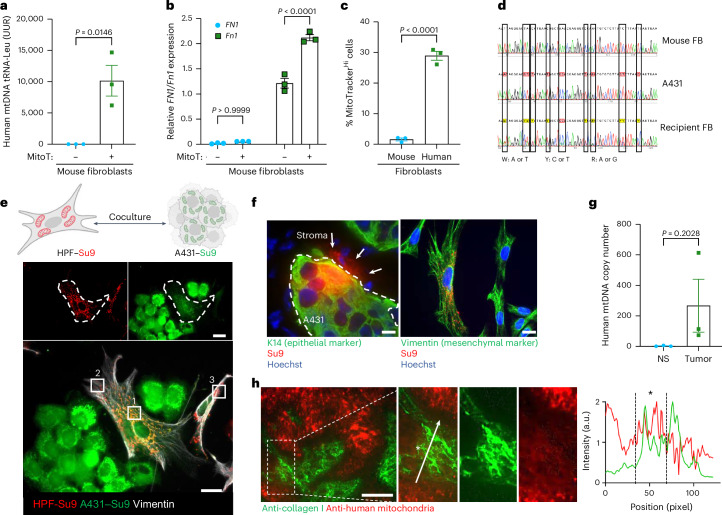
Cancer cells transfer mitochondria to fibroblasts in vitro and in vivo*.* **a**, qPCR for the human mtDNA encoding tRNA-Leu(UUR) relative to mouse nuclear DNA encoding beta2-microglobulin (B2m) using DNA from MitoTracker-positive and MitoTracker-negative mouse fibroblasts sorted from *n* = 3 cocultures. Total mtDNA content was calculated on the basis of *C*_*t*_ values. **b**, RT–qPCR for human and mouse *FN1* and *Fn1* relative to *RPL27* or *Rps29*, respectively, using RNA from MitoTracker-high and MitoTracker-low mouse fibroblasts sorted from *n* = 3 cocultures. **c**, Transfer efficiency in human–human and human–mouse cocultures (*n* = 3 cocultures per group). **d**, Comparison of SNPs within the 16S rRNA gene region of A431 cell mitochondria with those of control and recipient mouse fibroblasts. SNPs from A431 cells in recipient fibroblasts are indicated with rectangles (*n* > 300,000 cells pooled from three independent cocultures). **e**, Experimental setup and fluorescence images of HPF Su9–RFP and A431 Su9–GFP cocultures. This image was created with BioRender.com. Bottom, (1) colocalization of A431 and HPF mitochondria (orange), (2) HPF periphery with own mitochondrial network (red) and (3) nonrecipient HPFs (red). **f**, Section of a xenograft tumor formed by Su9–RFP A431 cells, showing Su9–RFP fluorescence in mitochondria of keratin 14 (K14)-negative cells and cultured fibroblasts from these tumors showing Su9–RFP fluorescence (red) and vimentin expression. **g**, qPCR for the mtDNA-encoded human tRNA-Leu gene relative to the mouse *B2m* gene using total DNA from cultured mouse fibroblasts isolated from noninjected ear skin (NS) or A431 xenograft tumors (*n* = 3 normal skin samples and *n* = 3 tumor samples from different mice). **h**, Representative immunofluorescence staining of A431 xenograft tumors for COLI (green) and human mitochondria (red). Costaining (yellow) of stromal cells adjacent to tumors was confirmed by colocalization analysis (site indicated with an asterisk). The white arrow indicates the line along which the intensity values of the different fluorescence signals were measured, starting from the initial position at the base of the arrow and ending at the arrowhead. Separate channels of zoomed-in regions are displayed (*n* = 3 sections from different tumors). Graphs show the mean ± s.e.m. An unpaired two-sided Student’s *t*-test (**a**,**c**,**g**) or two-sided one-way ANOVA with Bonferroni post hoc multiple comparison test (**b**) was used to determine statistical significance. One control value was set to 1. Scale bars, 25 μm (**e**,**f**,**h**). Source data

**Extended Data Fig. 1:**
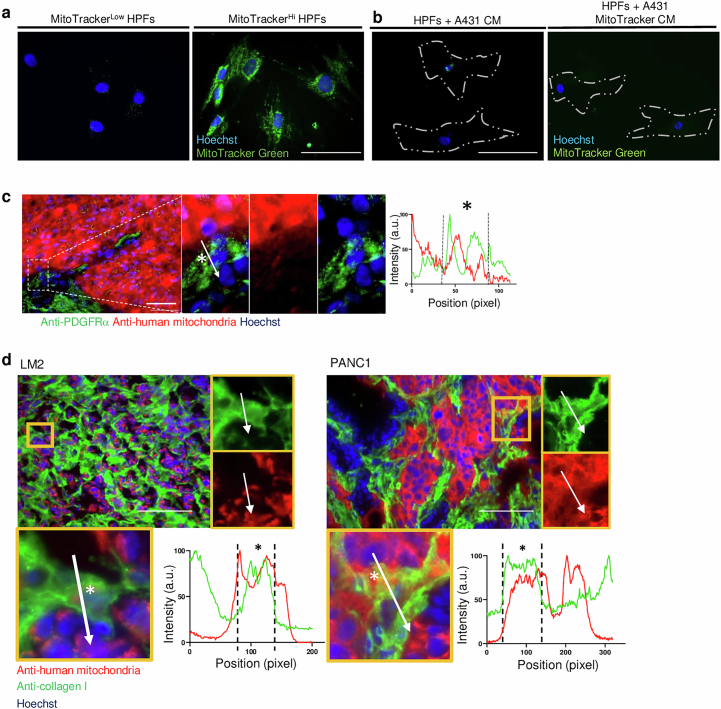
Different cancer cells transfer mitochondria to fibroblasts *in vivo.* **a**. Representative fluorescence images of FACS-sorted HPFs. Cells were sorted based on the intensity of MitoTracker Green staining, re-seeded for fluorescence analysis, and counterstained with Hoechst (blue). **b**. Representative fluorescence stainings of HPFs incubated with A431 CM (±MitoTracker Green staining) and counterstained with Hoechst (blue). **c**. Representative immunofluorescence staining of A431 skin xenograft sections for PDGFR-α (green) and an antibody specific for human mitochondria (red), co-stained with Hoechst (blue). Co-staining of stromal cells adjacent to tumors was confirmed by co-localization analysis (site indicated with an asterisk). White arrows in (**c**) and (**d**) indicate the line along which the intensity values of the different fluorescence signals were measured, starting from the initial position at the base of the arrow and ending at the arrowhead. Separate channels of zoomed-in regions are displayed. *n* = 3 sections from different tumors. **d**. Representative sections of breast and pancreatic cancer xenograft tumors from LM2 and PANC1 cells, respectively, immunostained for COLI (green) and co-stained with an antibody that specifically detects human mitochondria (red). Co-staining (yellow) of stromal cells adjacent to tumors was confirmed by co-localization analysis (site indicated with an asterisk). Separate channels of zoomed-in regions are displayed. *n* = 3 sections from different tumors. Scale bars: 100 μm (**a**–**d**). Source data

The selective effects of actin polymerization inhibitors and of SEC3–SEC5 small interfering RNAs (siRNAs) strongly suggest that the bright signal observed in some fibroblasts adjacent to cancer cells resulted from mitochondrial transfer rather than from dye leakage. Nevertheless, we performed additional controls to further confirm the specificity. Fluorescence analysis of fluorescence-activated cell sorting (FACS)-sorted HPFs showed that MitoTracker was stably incorporated into their mitochondrial network after serial passages and MitoTracker staining was not detectable after culture of HPFs in the conditioned medium (CM) of MitoTracker-treated A431 cells (Extended Data Fig. 1a,b). As an alternative, we used cocultures of human A431 cells and MitoTracker green-positive mouse fibroblasts. After 24 h, we detected human mitochondrial DNA in the fibroblasts by PCR (Fig. 2a). They exclusively expressed murine fibronectin 1 (*Fn1*), confirming their murine origin (Fig. 2b). Therefore, human cancer cells also transfer their mitochondria into mouse fibroblasts, although the transfer efficiency was significantly lower than in the human–human cocultures (Fig. 2c). We confirmed the transfer by making use of species-specific single-nucleotide polymorphisms (SNPs)^10^. Unique sequence variants within the 16S ribosomal RNA (rRNA) gene region of A431 cell mitochondria were detected in the mitochondria of the recipient mouse fibroblasts (Fig. 2d).

Next, we stably expressed mitochondria-targeted red fluorescent protein (Su9–RFP) in HPFs and green fluorescent protein (Su9–GFP) in A431 cells. After 24 h coculture, we detected HPFs with mitochondria that appeared orange in close proximity to the GFP-labeled cancer cells (Fig. 2e), confirming the uptake of mitochondria from cancer cells and suggesting their fusion with mitochondria of recipient fibroblasts. A431 cells expressing Su9–RFP were then injected intradermally into the ears of immunocompromised NOD scid mice. Fluorescence microscopy analysis of the resulting tumors showed mitochondrial structures of stromal fibroblasts that were positive for the mitochondrial protein expressed by cancer cells. We also detected human mitochondrial DNA (mtDNA) in cultured primary mouse fibroblasts from the tumors (Fig. 2f,g).

Lastly, we stained sections from skin cancer xenograft tumors formed by A431 cells^16^ with an antibody specific for human mitochondria. In addition to the expected strong staining of the tumor cells, adjacent stromal cells showed clear staining, which overlapped with staining for the pan-fibroblast markers collagen type I (COLI) or platelet-derived growth factor alpha (PDGFRα)^22^ (Fig. 2h and Extended Data Fig. 1c).

The transfer of mitochondria into fibroblasts in vivo was verified with breast and pancreatic cancer cells by costaining of respective xenograft tumors with antibodies to human mitochondria and COLI (Extended Data Fig. 1d).

### Mitochondrial transfer induces a CAF phenotype

**Fig. 3:**
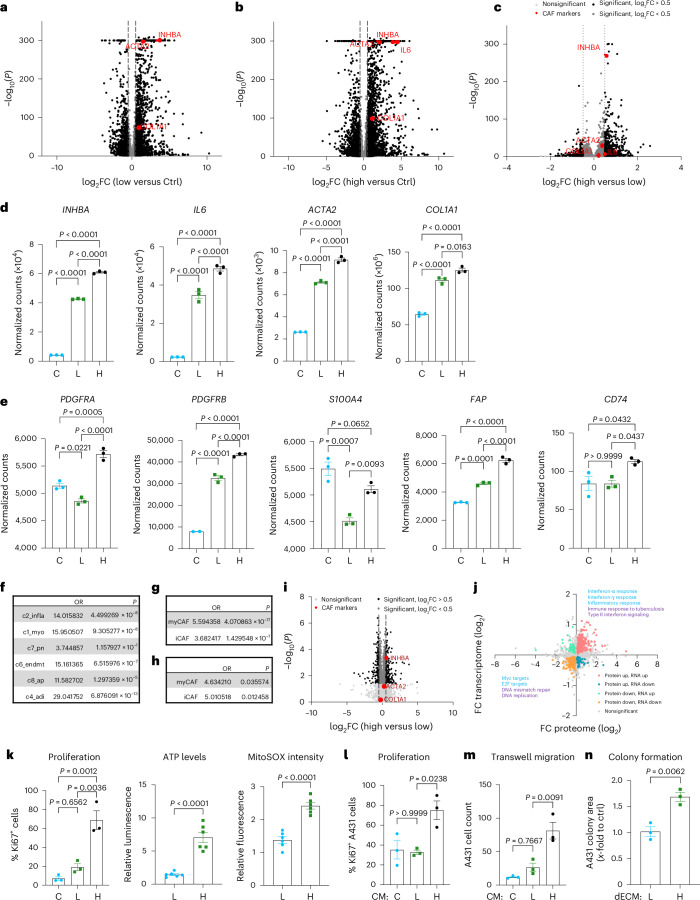
Transferred cancer cell mitochondria induce a CAF phenotype. **a**–**c**, Volcano plots displaying differentially expressed genes in MitoTracker-low versus control (**a**), MitoTracker-high versus control (**b**) and MitoTracker-high versus MitoTracker-low HPFs (**c**) sorted from *n* = 3 cocultures with A431 cells. **d**,**e**. RNA-seq data from sorted HPFs depicting expression of *INHBA*, *IL6*, *ACTA2* and *COL1A1* (**d**) or *PDGFRA*, *PDGFRB*, *S100A4*, *FAP* and *CD74* (**e**) in MitoTracker-high, MitoTracker-low and control groups sorted from *n* = 3 cocultures. **f**–**h**, Comparative analysis of gene signatures in MitoTracker-high versus MitoTracker-low HPFs with published CAF datasets^23,28,30^, showing similarities of MitoTracker-high HPFs with myCAFs and iCAFs. **i**, Volcano plot displaying differentially abundant proteins in MitoTracker-high versus MitoTracker-low HPFs sorted from *n* = 4 cocultures with A431 cells. **j**, Correlation analysis of gene and protein expression in MitoTracker-high versus MitoTracker-low HPFs. Significantly regulated pathways (*q* < 0.1) are highlighted (blue, Hallmarks of Cancer pathways; purple, Wikipathways). **k**, Percentage of Ki67-positive HPFs and relative levels of intracellular ATP and MitoSOX in sorted HPFs (*n* = 3 cocultures for Ki67 and 6 cocultures for ATP and MitoSOX). **l**, Percentage of Ki67-positive A431 cells in spheroids cultured with CM from control HPFs and sorted MitoTracker-high and MitoTracker-low HPFs (*n* = 3 spheroids per treatment group). **m**, Transwell migration of A431 cells in CM from sorted MitoTracker-low and MitoTracker-high and control HPFs (*n* = 3 transwell cultures per treatment group). **n**. Relative colony size of A431 cells plated on dECM from sorted HPFs (*n* = 3 cultures). Graphs show the mean ± s.e.m. An unpaired two-sided Student’s *t*-test (**k** (right and middle),**n**) or two-sided one-way ANOVA with Bonferroni post hoc multiple comparison test (**d**,**e**,**k** (left),**l**,**m**) was used to determine statistical significance. One control value was set to 1. Source data

**Extended Data Fig. 2:**
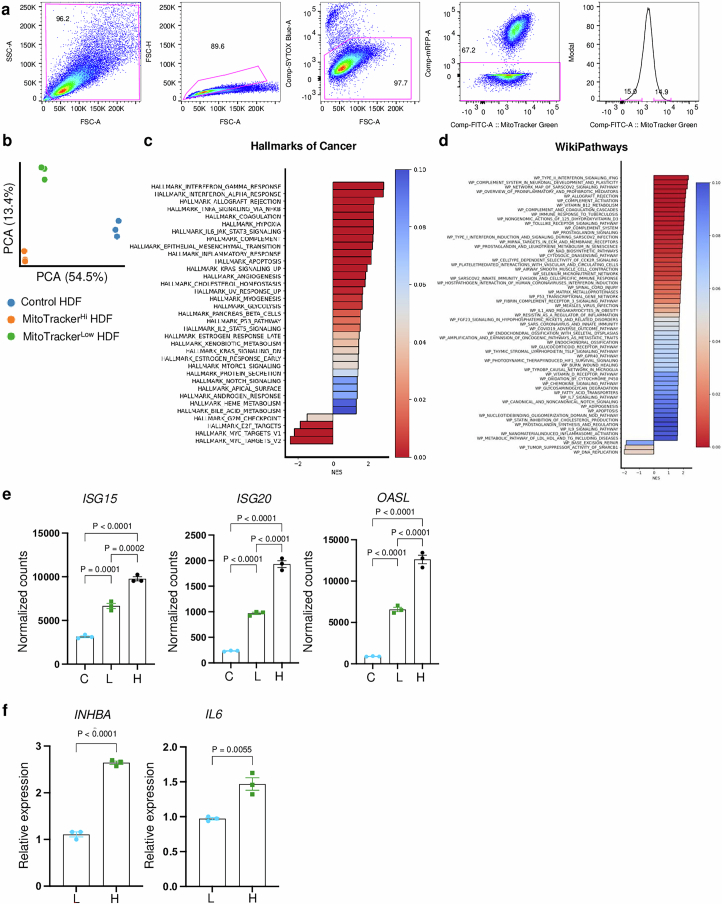
RNA-Seq data of MitoTracker-high and -low HPFs in co-cultures. **a**. FACS gating strategy for isolation of HPFs from A431-HPF co-cultures, demonstrating the arbitrary division into two HPF populations. FSC: Forward scatter; SSC; side scatter. **b**. Principal Component Analysis (PCA) of RNA-seq data from MitoTracker-high and -low HPFs and control HPFs that underwent the sorting procedure but were not maintained in co-cultures. *n* = 3 co-cultures per group. **c**, **d**. Volcano plots showing differentially expressed genes in HPFs in co-culture (MitoTracker-high (**c**) or -low (**d**)) *vs*. control HPFs in monoculture. Selected CAF marker genes are in red. *n* = 3 co-cultures and sorting experiments. **e**. RNA-seq data showing expression of ISGs in MitoTracker -high and -low HPFs after co-culture with A431 cells *vs*. control HPFs. *n* = 3 co-cultures per group. **f**. qRT-PCR for *INHBA and IL6* relative to *RPL27* using RNA from MitoTracker-high and -low HPFs after co-culture with A431 cells *vs*. control HPFs (repetition experiment). *n* = 3 co-cultures per group. Graphs show mean ± SEM. Unpaired two-sided Student’s t-test (**f**) or two-sided one-way ANOVA with Bonferroni post-host multiple comparison test (**e**) were used to determine statistical significance. Source data

To assess the functional relevance of the mitochondrial transfer, we sorted viable HPFs with high and low MitoTracker Green fluorescence intensity (Extended Data Fig. 2a) and analyzed them by RNA sequencing (RNA-seq). Control fibroblasts were subjected to the sorting procedure but not maintained in cocultures. E-cadherin mRNA was not detected in the sorted fibroblasts, confirming the efficient sorting. Principal component analysis (PCA) showed distinct clustering (Extended Data Fig. 2b). There were significant differences in gene expression between HPFs in the coculture (MitoTracker-high and MitoTracker-low) versus control HPFs in monoculture and also between MitoTracker-high and MitoTracker-low HPFs (Fig. 3a–c), although the two latter populations were exposed to the same cancer cell secretome. This finding suggests a strong impact of cancer-cell-derived mitochondria on the recipient HPFs. Genes significantly upregulated in the MitoTracker-high versus MitoTracker-low group were predominantly involved in pathways related to inflammation, immune response, cellular metabolism and stress responses (Extended Data Fig. 2c,d). Activation of the interferon pathway was reflected by the increased expression of several interferon response genes (ISGs) (Extended Data Fig. 2e).

Because the pathways enriched in the MitoTracker-high fibroblasts are often activated in CAFs^23–25^, we analyzed the dataset for skin CAF marker genes^24,26–28^. Many of them were indeed overexpressed in the MitoTracker-high and, to a lesser extent, in the MitoTracker-low population. Among them were *INHBA*^16^ (encoding the protumorigenic cytokine activin A), *IL6* (encoding interleukin-6), *ACTA2* (encoding α smooth muscle actin) and *COL1A1* (encoding COLI, alpha 1 subunit) (Fig. 3d). This was verified by reverse transcription (RT)–qPCR in independent coculture experiments (Extended Data Fig. 2f). *PDGFRA* and the CAF markers *PDGFRB*, *S100A4* (encoding FSP1), *FAP* and *CD74* (ref. ^29^) were also upregulated in the MitoTracker-high population (Fig. 3e).

To determine whether the MitoTracker-high cells (Mito-CAFs), correspond to inflammatory CAFs (iCAFs), myofibroblastic CAFs (myCAFs) or antigen-presenting CAFs (apCAFs)^29^, we compared their expression profile to those of published CAF datasets^23,28,30^. Mito-CAFs overexpressed genes characteristic of both iCAFs and myCAFs (Fig. 3f–h).

**Extended Data Fig. 3:**
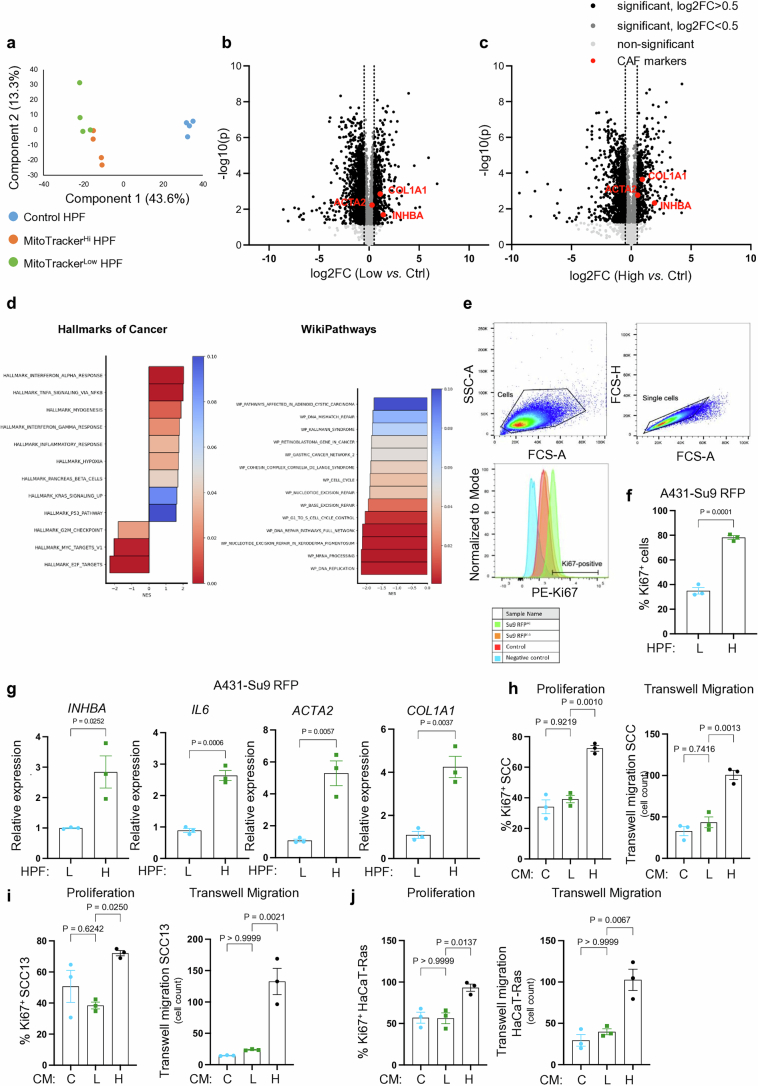
Proteomics analysis of HPFs in co-cultures and CAF features of recipient fibroblasts. **a**. PCA of proteomics data from MitoTracker-high and -low and control HPFs. *n* = 4 co-cultures and sorting experiments. **b**. Volcano plot showing differentially abundant proteins in sorted MitoTracker-low *vs*. control HPFs. *n* = 4 co-cultures per group. Selected CAF marker proteins are in red. **c**. Volcano plot showing differentially abundant proteins in sorted MitoTracker-high *vs*. HPFs. *n* = 4 co-cultures per group. Selected CAF marker proteins are in red. **d**. Unbiased pathway analysis of MitoTracker-high *vs*. - low HPFs referenced to Hallmarks of Cancer (left) and WikiPathways (right). **e**. Gating strategy (top panel) and representative histogram of flow cytometry for Ki67^+^ cells among RFP-high and -low and control HPFs (bottom panel). Gating was set based on the negative control. **f**. Percentage of Ki67^+^ cells among RFP-high- and -low HPFs after co-culture with A431 cells expressing Su9-RFP. *n* = 3 co-cultures per group. **g**. qRT-PCR for *INHBA*, *IL6*, *ACTA2* and *COL1A1* relative to *RPL27* using RNA from RFP–high and -low HPFs after co-culture with A431 cells expressing Su9-RFP. *n* = 3 co-cultures per group. **h**, **j**. Percentage of Ki67^+^ cells and transwell migration of primary cutaneous SCC cells (**h**), SCC13 cells (**i**) and HaCaT-Ras cells (**j**) cultured with CM from control or MitoTracker-low or -high HPFs sorted after co-culture with the respective cancer cell lines. *n* = 3 co-cultures per group. Graphs show mean ± SEM. Unpaired two-sided Student’s t-test (**f**, **g**) or two-sided one-way ANOVA with Bonferroni post-host multiple comparison test (**h**–**j**) were used to determine statistical significance. Source data

A proteomic analysis using the same fibroblast populations revealed clear clustering of the groups (Extended Data Fig. 3a), increased abundance of CAF markers and ISG-encoded proteins and activation of proinflammatory pathways in the MitoTracker-high population (Fig. 3i, Extended Data Fig. 3b–d and Supplementary Table 1). Many of the observed gene expression changes were reflected by protein abundance changes (Fig. 3j).

MitoTracker-high fibroblasts also exhibited functional characteristics of protumorigenic CAFs, including increased proliferation and higher concentrations of intracellular adenosine triphosphate (ATP) and mitochondrial reactive oxygen species (ROS) (Fig. 3k)^14,16^.

Increased proliferation and CAF marker expression in sorted fibroblasts were also observed when HPFs in cocultures received mitochondria from A431 cells expressing Su9–RFP (Extended Data Fig. 3e–g), further confirming the reliability of the MitoTracker approach in our setting.

Lastly, the CM from MitoTracker-high HPFs promoted cancer cell proliferation and transwell migration more efficiently than CM from MitoTracker-low HPFs. This was observed for A431 cells (Fig. 3l,m), primary skin squamous cell carcinoma (SCC) cells and SCC13 (ref. ^31^) and HA–Ras-transformed HaCaT cells (HaCaT-Ras)^32^ (Extended Data Fig. 3h–j). Furthermore, the decellularized extracellular matrix (dECM) produced by MitoTracker-high fibroblasts induced the formation of larger A431 colonies (Fig. 3n).

### Mitochondrial transplantation induces CAF reprogramming

**Fig. 4:**
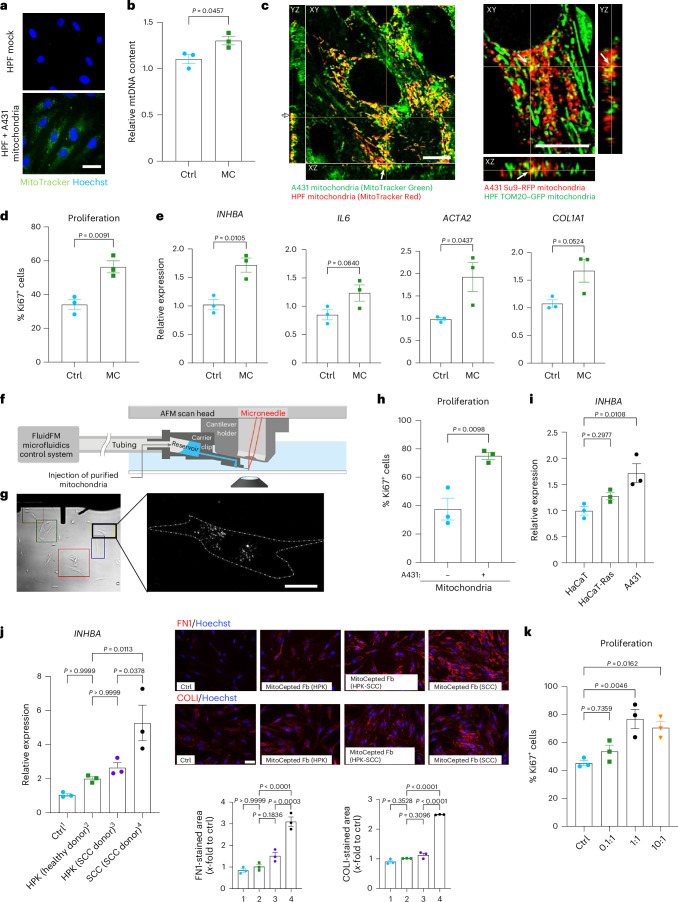
CAF reprogramming through transplantation of cancer cell mitochondria. **a**, Representative fluorescence images of HPFs MitoCepted with MitoTracker green-stained A431 mitochondria (MitoCepted HPFs) or mock treatment, counterstained with Hoechst (blue) (*n* = 3 cultures per group). **b**, qPCR for the mtDNA encoding tRNA-Leu(UUR) relative to the nucDNA encoding B2M using DNA from MitoCepted (MC) or mock-treated (Ctrl) HPFs. Relative mtDNA content (based on *C*_*t*_ values) is indicated (*n* = 3 cultures per group). **c**, Representative confocal image in the *xyz* plane showing HPFs prestained with MitoTracker red and MitoCepted with MitoTracker green-labeled A431 mitochondria (left) and TOM20–GFP-expressing HPFs MitoCepted with mitochondria from A431 Su9–RFP cells (right). Yellow staining indicates mitochondrial fusion. **d**, Percentage of Ki67^+^ MitoCepted or control HPFs among all cells (*n* = 3 cultures per group). **e**, RT–qPCR for *INHBA*, *IL6*, *ACTA2* and *COL1A1* relative to *RPL27* using RNA from MitoCepted or mock-treated HPFs (*n* = 3 cultures per group). **f**, FluidFM experimental setup, adapted from a previous study^36^. **g**, Image of FluidFM-mediated injection of mitochondria into HPFs. **h**, Percentage of Ki67^+^ fibroblasts in HPFs injected with A431-derived mitochondria using FluidFM or mock treatment (*n* = 3 cultures per group). **i**, RT–qPCR for *INHBA* using RNA from HPFs subjected to MitoCeption with mitochondria from HaCaT, HaCaT-Ras or A431 cell lines (*n* = 3 cultures per cell line). **j**, RT–qPCR for *INHBA* using RNA from (1) mock-treated HPFs or HPFs subjected to MitoCeption with mitochondria (2) from keratinocytes of a healthy individual, (3) from normal keratinocytes of a person with SCC or (4) malignant cancer cells of the same person with SCC. Right, representative FN1–COLI immunofluorescence stainings with quantification of staining intensity in the dECM produced by HPFs after MitoCeption with mitochondria from the different primary donor cells (*n* = 3 MitoCeptions per cell type). **k**, Percentage of Ki67^+^ HPFs subjected to MitoCeption with different amounts of mitochondria isolated from A431 cells. Numbers on the *x* axis show the ratio of donor A431 cells (used for mitochondrial isolation) and recipient HPFs (*n* = 3 cultures per group). Graphs show the mean ± s.e.m. An unpaired two-sided Student’s *t*-test (**b**,**d**,**e**,**h**) or two-sided one-way ANOVA with Bonferroni post hoc multiple comparison test (**i**–**k**) was used to determine statistical significance. Scale bars, 100 μm (**a**,**j**), 10 μm (**c**) and 25 μm (**g**). Source data

To specifically test the role of cancer cell mitochondria in fibroblast reprogramming, we isolated and purified mitochondria from cancer cells and transplanted them directly into HPFs using MitoCeption^33^. While mitochondria directly move from the cytoplasm of the donor cells to the cytoplasm of recipient cells during TNT-mediated transfer, MitoCeption induces the rapid uptake of purified mitochondria through the plasma membrane, most likely through an endocytic pathway^33^. The uptake using MitoCeption was confirmed by detection of MitoTracker green fluorescence and by an increase in mtDNA content in the ‘MitoCepted’ fibroblasts (Fig. 4a,b), which was in a similar range to that described for MitoCepted endothelial cells (13%)^5^. The MitoTracker staining likely overestimates the uptake because cancer cell mitochondria fuse with the mitochondria of recipient HPFs, as seen after transplantation of MitoTracker green-labeled or Su9–RFP-expressing mitochondria from A431 cells into HPFs prestained with MitoTracker red or expressing TOM20–GFP, respectively (Fig. 4c). We found a substantial colocalization of the MitoCepted and the endogenous mitochondria using confocal microscopy. As expected, it was more pronounced with MitoTracker green because the dye labels the entire mitochondria. Although an additional effect of dye leakage cannot be excluded, the findings obtained with MitoTracker-labeled and, in particular, with genetically labeled mitochondria demonstrate that the MitoCepted mitochondria were released into the cytoplasm.

**Extended Data Fig. 4:**
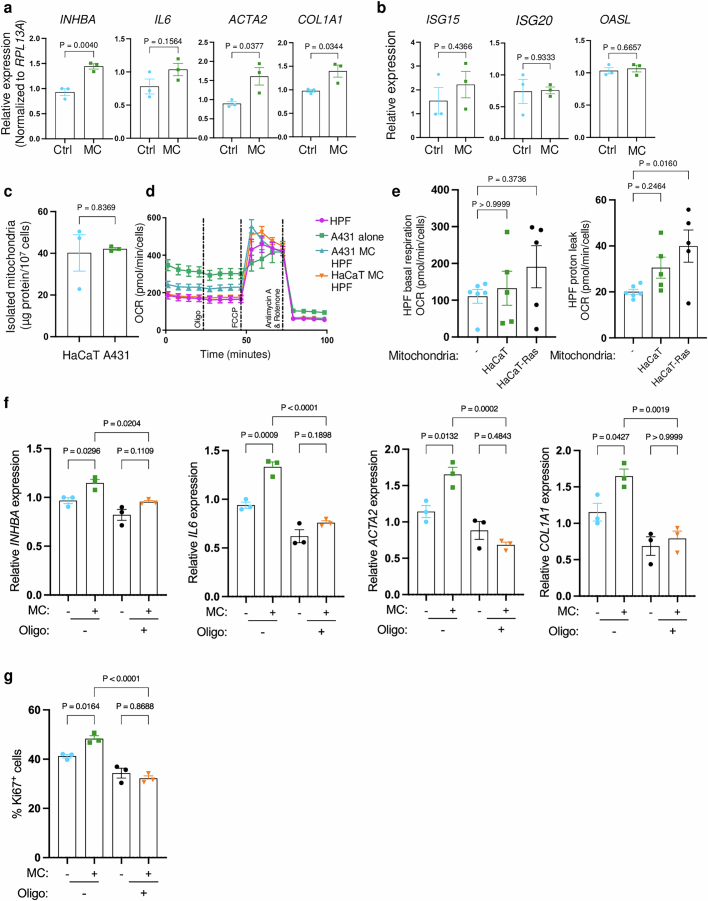
HPFs acquire CAF properties after MitoCeption with cancer cell mitochondria. **a**. qRT-PCR for *INHBA, IL6, COL1A1* and *ACTA2* relative to *RPL13A* using RNA from MitoCepted (A431 mitochondria) or mock-treated HPFs. *n* = 3 cultures per group. **b**. qRT-PCR for *ISG15, ISG20*, and *OASL* relative to *RPL27* using RNA from MitoCepted or mock-treated HPFs. *n* = 3 cultures per group. **c**. BCA protein quantification in mitochondrial lysates from 10^7^ HaCaT or A431 cells. *n* = 3 cultures per cell line. **d**. Seahorse analysis of HPFs subjected to MitoCeption with A431 or HaCaT mitochondria or mock treatment, and of A431 cells. *n* = 5 cultures per group. **e**. Basal respiration and proton leak of HPFs, subjected to MitoCeption with HaCaT or HaCaT-Ras mitochondria or mock treatment, determined by Seahorse XF stress test. *n* = 5 cultures per group. **f**. qRT-PCR for *INHBA, IL6, COL1A1* and *ACTA2* relative to *RPL27* using RNA from HPFs subjected to MitoCeption with MitoTracker Green-stained mitochondria from A431 cancer cells, pre-treated with oligomycin or mock treatment. *n* = 3 cultures per group. **g**. Percentage of Ki67-positive HPFs subjected to MitoCeption with MitoTracker Green-stained mitochondria from A431 cancer cells, pre-treated with oligomycin or mock treatment. *n* = 3 cultures per treatment group. Graphs show mean ± SEM. Unpaired two-sided Student’s t-test (**a**–**c**) or two-sided one-way ANOVA with Bonferroni post-host multiple comparison test (**e**–**g**) were used to determine statistical significance. Source data

We found that, 24 h after MitoCeption with MitoTracker-labeled A431 mitochondria, the proliferation rate was significantly higher compared to mock-treated HPFs (Fig. 4d) and the MitoCepted cells showed higher expression of CAF genes (Fig. 4e and Extended Data Fig. 4a). By contrast, expression of three selected ISGs was not increased after MitoCeption (Extended Data Fig. 4b), suggesting that mitochondrial transfer alone is not sufficient to activate these genes.

To validate the effect of purified mitochondria on CAF differentiation, we used fluid force microscopy (FluidFM)^34–36^ to inject purified MitoTracker Green-stained A431 mitochondria directly into the cytoplasm of HPFs (Fig. 4f,g). The injected HPFs also showed increased proliferation (Fig. 4h), again demonstrating that the effect of mitochondria on the induction of CAF features is independent of the mode of uptake.

We next compared the effect of mitochondria isolated from the HaCaT keratinocyte cell line, their malignant counterpart HaCaT-Ras and the highly malignant A431 cell line on HPFs in MitoCeption experiments using mitochondria isolated from the same number of cells. This normalization is justified because of the similar protein content of the mitochondrial isolates from all cell lines (Extended Data Fig. 4c). The expression of *INHBA* in the recipient HPFs increased in accordance with the malignancy of the donor cell line (Fig. 4i). Consistently, MitoCepted fibroblasts, which received mitochondria from primary donor-derived SCC cells, displayed increased expression of *INHBA* compared to fibroblasts, which received mitochondria from adjacent nontransformed keratinocytes or from keratinocytes of a healthy individual, and deposited more FN1 and COLI (Fig. 4j).

The effect of mitochondria on HPFs was concentration dependent. MitoCeption of HPFs with A431 mitochondria at a 1:1 ratio (same number of donor A431 cells and recipient HPFs) caused a significant increase in HPF proliferation, while lower amounts of mitochondria had only a minor effect. A further increase in the amount of MitoCepted mitochondria did not further promote HPF proliferation (Fig. 4k).

### Functional cancer cell mitochondria induce CAF reprogramming

**Fig. 5:**
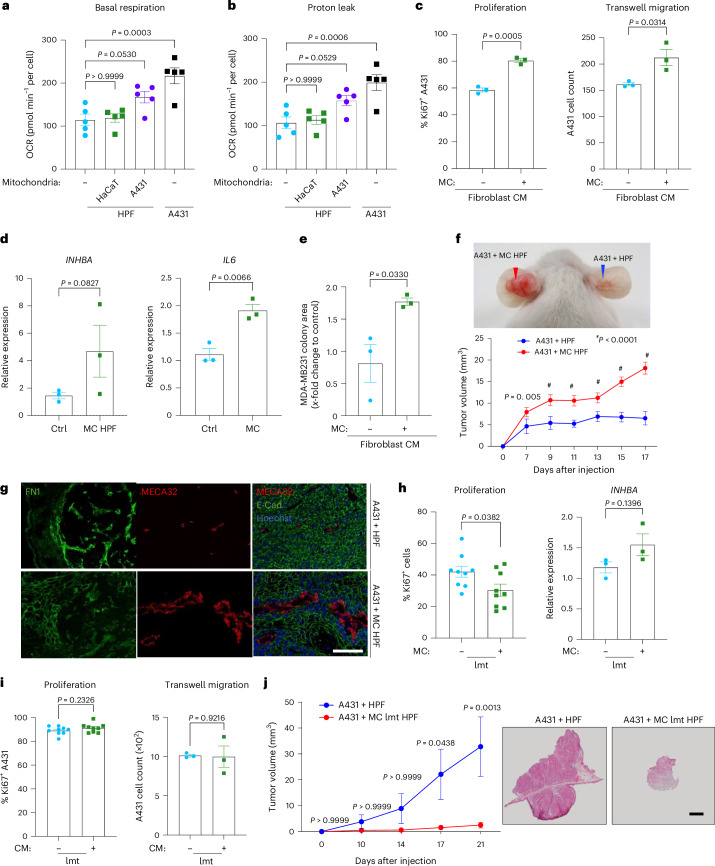
Functional cancer cell mitochondria are required for CAF reprogramming. **a**, Basal respiration of HPFs subjected to MitoCeption with A431 or HaCaT mitochondria or mock treatment in comparison to A431 cells (*n* = 5 independent MitoCeptions per cell type). **b**, Proton leak in the same cultures as in **a**. **c**, Percentage of Ki67^+^ A431 cells and transwell migration of A431 cells cultured in CM of MitoCepted or mock-treated HPFs (*n* = 3 cultures per group). **d**, RT–qPCR for *INHBA* and *IL6* using RNA from MitoCepted (mitochondria from MDA-MB-231 breast cancer cells) or mock-treated HPFs (*n* = 3 cultures per group). **e**, Clonogenicity of MDA-MB-231 cells cultured in CM from HPFs subjected to MitoCeption with MitoTracker green-stained mitochondria from MDA-MB-231 cells or mock treatment (*n* = 3 cultures per treatment group). **f**, Representative image of 3-week-old ear xenograft tumors (arrowheads) following intradermal coinjection of A431 cells and MitoCepted (with A431 mitochondria) or mock-treated HPFs and tumor volume at various time points (*n* = 5 tumors per group from different mice). **g**, Representative immunofluorescence stainings of tumors formed by A431 cells and MitoCepted or mock-treated HPFs for E-cadherin and FN1 (green) and MECA32 (red), counterstained with Hoechst (blue) (*n* = 5 tumors per group from different mice). **h**, Percentage of Ki67^+^ HPFs subjected to MitoCeption with A431 lmt mitochondria or mock treatment and RT–qPCR for *INHBA* using RNA from HPFs subjected to MitoCeption with A431 lmt mitochondria or mock treatment (*n* = 9 Ki67 or *n* = 3 RT–qPCR cultures per treatment group). **i**, Percentage of Ki67^+^ A431 cells (left) or transwell migration of A431 cells (right) cultured in CM from HPFs subjected to MitoCeption with A431 lmt mitochondria or mock treatment (*n* = 9 Ki67 or *n* = 3 transwell migration cultures per treatment group). **j**, Left, tumor volume at various time points during tumor development by A431 cancer cells coinjected with MitoCepted HPFs, which received mitochondria from control or lmt A431 cells (*n* = 4 tumors per group from different mice). Right, Histological stainings of a tumor from each group. Graphs show the mean ± s.e.m. An unpaired two-sided Student’s *t*-test (**c**–**e**,**h**,**i**) or two-sided one-way (**a**,**b**) or two-way (**f**,**j**) ANOVA with Bonferroni post hoc multiple comparison test was used to determine statistical significance. Scale bars, 200 μm (**g**) and 1 mm (**j**). Source data

To gain mechanistic insight into the alterations in HPFs that occur upon mitochondrial transfer from cancer cells, we measured their oxygen consumption rate (OCR) using Seahorse analysis. Transplantation of A431-derived but not HaCaT cell-derived mitochondria promoted basal respiration and proton leak in the recipient fibroblasts (Fig. 5a,b and Extended Data Fig. 4d). The values observed in HPFs after MitoCeption with A431 mitochondria almost reached those observed in A431 cells (Fig. 5a,b). Mitochondria from HaCaT-Ras cells also promoted proton leak but had no effect on basal respiration (Extended Data Fig. 4e). These findings provide a possible explanation for the higher proliferation of recipient HPFs because increased oxidative phosphorylation (OxPhos) in cultured fibroblasts was shown to promote their proliferation^37^. Consistently, inhibition of OxPhos in HPFs by oligomycin prevented the increase in CAF marker expression and proliferation after transplantation of A431 mitochondria (Extended Data Fig. 4f,g). High OCR and ATP levels in CAFs are also important for their release of protumorigenic factors^16^. Consistently, CM from fibroblasts, which received A431 mitochondria using MitoCeption, promoted proliferation and transwell migration of A431 cells to a significantly higher extent compared to CM from control (mock-treated) fibroblasts (Fig. 5c).

**Extended Data Fig. 5:**
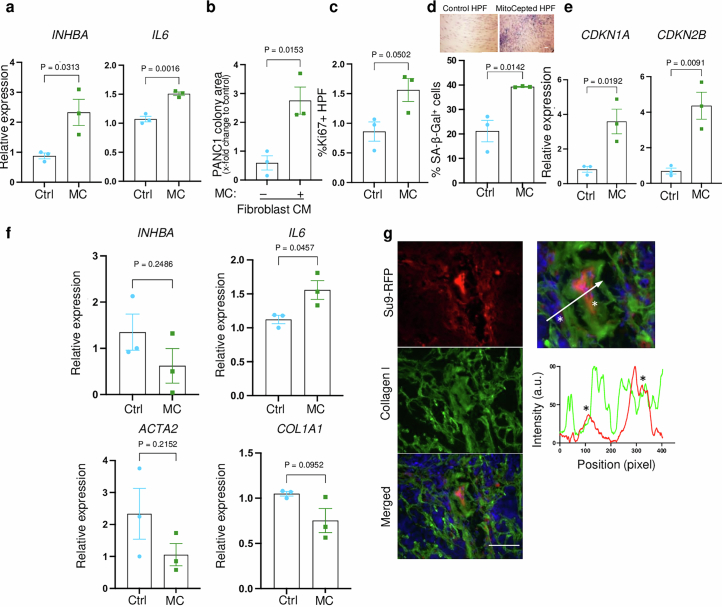
HPFs undergo senescence five days post-MitoCeption. **a**. qRT-PCR for *INHBA* and *IL6* using RNA from MitoCepted (mitochondria from PANC1 pancreatic cancer cells) or mock-treated HPFs. *n* = 3 cultures per group. **b**. Clonogenicity of PANC1 cells cultured in CM from HPFs subjected to MitoCeption with MitoTracker Green-stained mitochondria from PANC1 pancreatic cancer cells or mock treatment. *n* = 3 cultures per group. **c**. Percentage of Ki67-positive HPFs cultured for 5 days after MitoCeption with A431 mitochondria or mock treatment. *n* = 3 cultures per group. **d**. Representative photomicrographs of HPFs cultured for 5 days after MitoCeption with A431 mitochondria or mock treatment and stained for SA-β-Gal, and quantification of the percentage of SA-β-Gal positive cells. *n* = 3 cultures per group. **e**. qRT-PCR for *CDKN1A* and *CDKN2B* using RNA from HPFs subjected to MitoCeption with mitochondria from A431 cancer cells or mock treatment; 5 days after MitoCeption. *n* = 3 cultures per treatment group. **f**. qRT-PCR for *INHBA, IL6, COL1A1* and *ACTA2* using RNA from HPFs subjected to MitoCeption with MitoTracker Green-stained mitochondria from A431 cancer cells or mock treatment; 5 days after MitoCeption. *n* = 3 cultures per group. **g**. Representative xenograft tumor sections (two weeks after injection) showing MitoCepted Su9-RFP-positive fibroblasts that had received mitochondria from A431-Su9-RFP A431 cells. Co-localization analysis reveals the presence of Su9-RFP-positive mitochondria in collagen I-positive fibroblasts (indicated with an asterisk). The white arrow indicates the line along which the intensity values of the different fluorescence signals were measured, starting from the initial position at the base of the arrow and ending at the arrowhead. *n* = 3 sections from different tumors. Graphs show mean ± SEM. Unpaired two-sided Student’s t-test (**a**–**f**) was used to determine statistical significance. Scale bars: 20 μm (**d**), 100 μm (**g**). Source data

Together, these results suggest that the transfer or transplantation of epithelial cancer cell-derived mitochondria alone is sufficient to reprogram fibroblasts. This is not limited to SCC cells, as transfer of mitochondria from the breast and pancreatic cancer cell lines MDA-MB-231 and PANC1, respectively, also increased the expression of CAF markers and their CM promoted clonogenic growth of MDA-MB-231 and PANC1 cells (Fig. 5d,e and Extended Data Fig. 5a,b).

At day 5 after seeding, the proliferation rate of HPFs declined, particularly after MitoCeption (Extended Data Fig. 5c versus Fig. 4d). Concomitantly, the number of β-galactosidase-positive fibroblasts increased among the MitoCepted fibroblasts, indicating senescence (Extended Data Fig. 5d). This is supported by their increased expression of the senescence markers *CDKN1A* and *CDKN2B*, while expression of most CAF markers was no longer increased at this time point (Extended Data Fig. 5e,f). The only exception was *IL6*, which is also a senescence marker. Nevertheless, the recipient HPFs may still exert protumorigenic effects because senescent cells often have a protumorigenic senescence-associated secretory phenotype^38^. Indeed, when we coinjected A431-MitoCepted or mock-treated HPFs with A431 cells into the ears of immunodeficient mice, the tumors that formed in the presence of fibroblasts containing A431-derived mitochondria were significantly larger and showed increased deposition of FN1 and more blood vessels (Fig. 5f,g). This was associated with the long-term presence of the MitoCepted fibroblasts as determined in a separate experiment with HPFs, which received Su9–RFP-expressing A431 cancer cell mitochondria. Then, 2 weeks after injection, these tagged fibroblasts were still detectable (Extended Data Fig. 5g).

**Extended Data Fig. 6:**
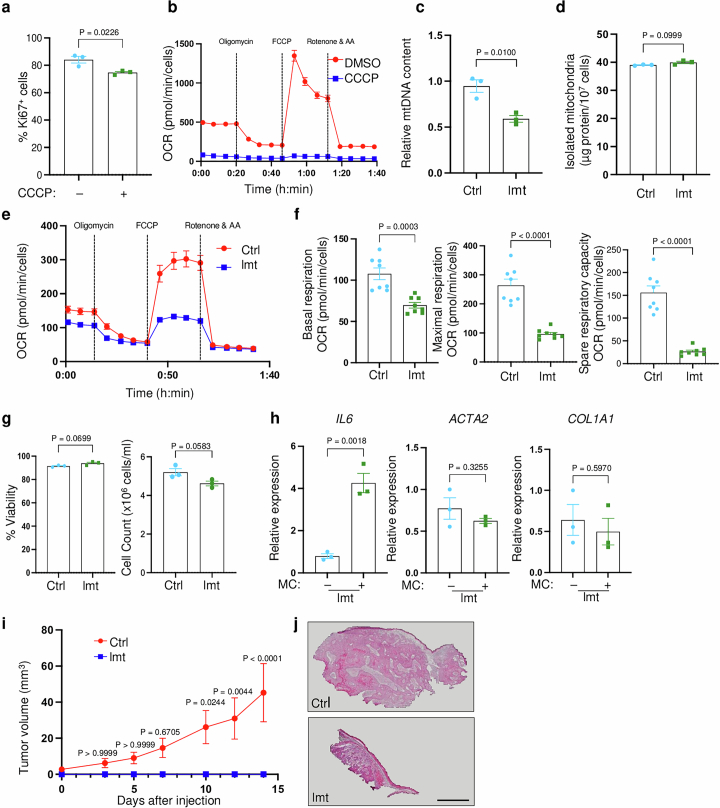
Functional cancer cell mitochondria are required to induce CAF properties. **a**. Percentage of Ki67-positive HPFs subjected to MitoCeption with mitochondria isolated from A431 cells pre-treated with CCCP or vehicle. *n* = 3 cultures per treatment group. **b**. Seahorse analysis of HPFs subjected to MitoCeption with mitochondria isolated from A431 cells pre-treated with CCCP (*n* = 12 cultures) or DMSO (*n* = 5 cultures). **c**. qPCR for the mtDNA encoding tRNA-Leu relative to nucDNA encoding B2 microglobulin using total DNA from A431 control or low mtDNA (lmt) cells. The relative mtDNA content was calculated based on the Ct values. *n* = 3 cultures per group. **d**. BCA protein quantification in mitochondrial lysates from 10^7^ A431 Ctrl and A431 lmt cells. *n* = 3 cultures per group. **e**. Oxygen consumption rate (OCR) of A431 Ctrl and A431 lmt cells, determined by Seahorse XF stress test. The time of drug injection is indicated. *n* = 8 cultures per group. **f**. Basal and maximal respiration and spare respiratory capacity of A431 Ctrl and A431 lmt cells. *n* = 8 cultures per group. **g**. Viability and cell count of A431 Ctrl and A431 lmt cells. *n* = 3 cultures per group. **h**. qRT-PCR for *IL6, COL1A1* and *ACTA2* relative to *RPL27* using RNA from HPFs subjected to MitoCeption with mitochondria from A431 Ctrl or A431 lmt cells. *n* = 3 cultures per group. **i**. Tumor volume at various time points during development of tumors formed by control or lmt (low mtDNA) A431 cells. *n* = 5 tumors per group from different mice. **j**. Representative images of H&E stainings of tumors from each group. *n* = 5 tumors per group from different mice. Scale bar: 1 mm. Graphs show mean ± SEM. Unpaired two-sided Student’s t-test (**a**, **c**, **d**, **f**–**h**) or two-sided two-way ANOVA with Bonferroni post-host multiple comparison test (**i**) were used to determine statistical significance. Scale bar: 1 mm (**j**). Source data

To test whether disruption of mitochondrial function in cancer cells prevents CAF differentiation upon mitochondrial transfer, we used FluidFM to extract mitochondria from A431 Su9–RFP cells, which were depolarized using carbonyl cyanide *m*-chlorophenylhydrazone (CCCP). HPFs, which received CCCP-treated mitochondria, had a mildly but significantly lower proliferation rate compared to HPFs, which received mock-treated mitochondria (Extended Data Fig. 6a). In addition, HPFs MitoCepted with mitochondria from A431 cells, which were pretreated with CCCP, had a strongly reduced OCR compared to HPFs that received mitochondria from vehicle-treated cancer cells (Extended Data Fig. 6b).

We next generated A431 cancer cells with a 40% reduction in the amount of mtDNA (termed low mtDNA (lmt) cells) using extended low-dose ethidium bromide treatment^39^ (Extended Data Fig. 6c). Mitochondria from lmt cells had a similar protein content to those from control cells and the viability and proliferation of lmt A431 cells were not reduced. However, their mitochondrial respiration was significantly impaired (Extended Data Fig. 6d–g). Upon transplantation of these mitochondria, the proliferation rate of the recipient cells was even reduced and expression of most CAF markers was not significantly altered (Fig. 5h and Extended Data Fig. 6h). The CM of HPFs, which received mitochondria from lmt cancer cells, did not promote proliferation and migration of cancer cells (Fig. 5i). In xenograft experiments, tumors formed by A431 cells coinjected with HPFs containing mitochondria from lmt A431 cells were significantly smaller compared to those formed with HPFs containing mitochondria from control A431 cells (Fig. 5j). This finding underscores the critical role of mitochondrial DNA, which encodes important components of the respiratory chain, in the induction of a protumorigenic CAF phenotype in MitoCepted fibroblasts. Additional experiments using only lmt A431 cells showed that mice injected with these cells did not develop tumors within 15 days (Extended Data Fig. 6i,j).

### MIRO2 is expressed in invasively growing cancer cells

**Fig. 6:**
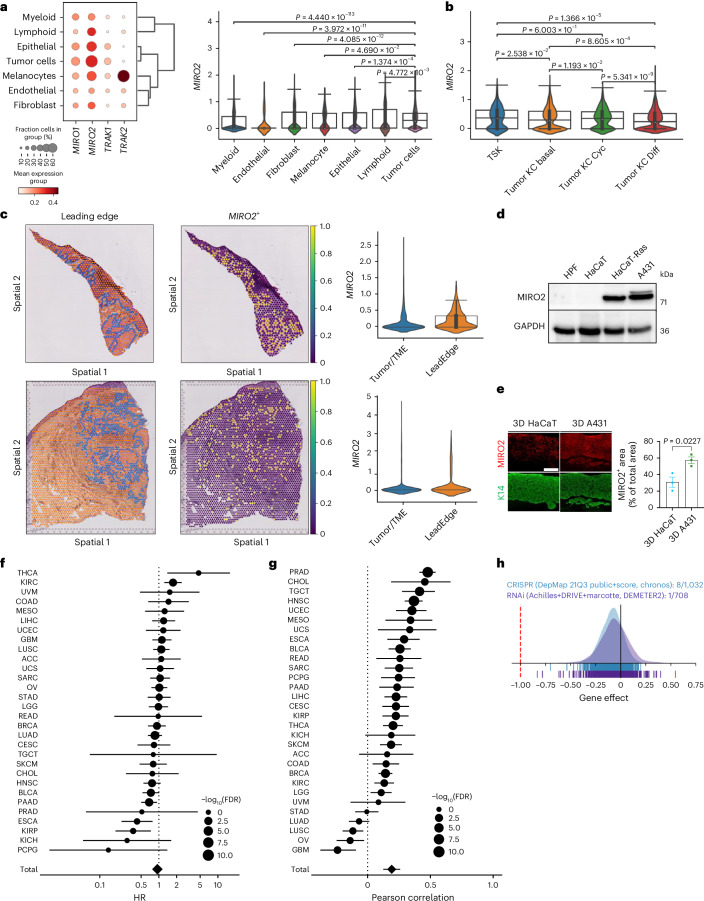
*MIRO2* is overexpressed at the leading edge of SCCs. **a**, Dot plot showing expression of mitochondrial trafficking genes; violin plot showing expression of *MIRO2* in different cell types in SCCs (*n* = 5,799 myeloid cells, 4,644 tumor cells, 1,495 epithelial cells, 584 fibroblasts, 413 lymphoid cells, 169 endothelial cells and 129 melanocytes). **b**, Violin plot showing expression of *MIRO2* in tumor cell subpopulations in SCCs based on scRNA-seq data^40^ (*n* = 296 tumor-specific keratinocytes (TSKs), 1,385 basal tumor keratinocytes (KC), 725 cycling tumor keratinocytes and 2238 differentiating tumor keratinocytes). **c**, Feature plots showing spatial distribution of MIRO2 transcripts in human skin SCC; violin blots showing MIRO2 transcripts at the tumor leading edge versus the total tumor and its microenvironment (TME) (*n* = 2 tumors from different patients; P2 and P6)^40^. **d**, Western blot of lysates from HPFs, HaCaT, HaCaT-Ras and A431 cells for MIRO2 and GAPDH. **e**, MIRO2 and K14 immunofluorescence stainings of sections from 3D organotypic skin cultures with HPFs and HaCaT or A431 cells and quantification of the MIRO2-positive area (*n* = 3 3D cultures per epithelial cell line). Scale bar, 100 μm. **f**, Forest plot showing the 5-year disease-specific survival (DSS) associated with *MIRO2* expression across solid cancers based on TCGA. Hazard ratios (HRs) and 95% confidence intervals (CIs) based on Cox proportional hazard model are shown. The last point represents the estimate from the random-effects meta-analysis (*n* = 8,941 patients). **g**, Pearson correlation coefficient (ρ) and 95% CIs between the enrichment score of the leading edge (LE) signature and *MIRO2* expression across the different solid cancers in TCGA. The last point represents the estimate from the random-effects meta-analysis (*n* = 10,238 patients). **h**, Dependency of different cancers on *MIRO2* expression as documented in the DepMap Portal. Gene effect scores are derived from DEMETER2 or CERES. A lower score denotes a greater dependency on expression. Violin plots in **a**–**c** show the median (center line), 25th and 75th percentiles (box bounds) and whiskers extending to the most extreme data points within 1.5 times the interquartile range from the box. Points outside this range are plotted as outliers. The graph in **e** shows the mean ± s.e.m. A Mann–Whitney *U*-test for comparison between two groups (**a**,**b**) or unpaired two-sided Student’s *t*-test (**e**) was used to determine statistical significance. Source data

**Extended Data Fig. 7:**
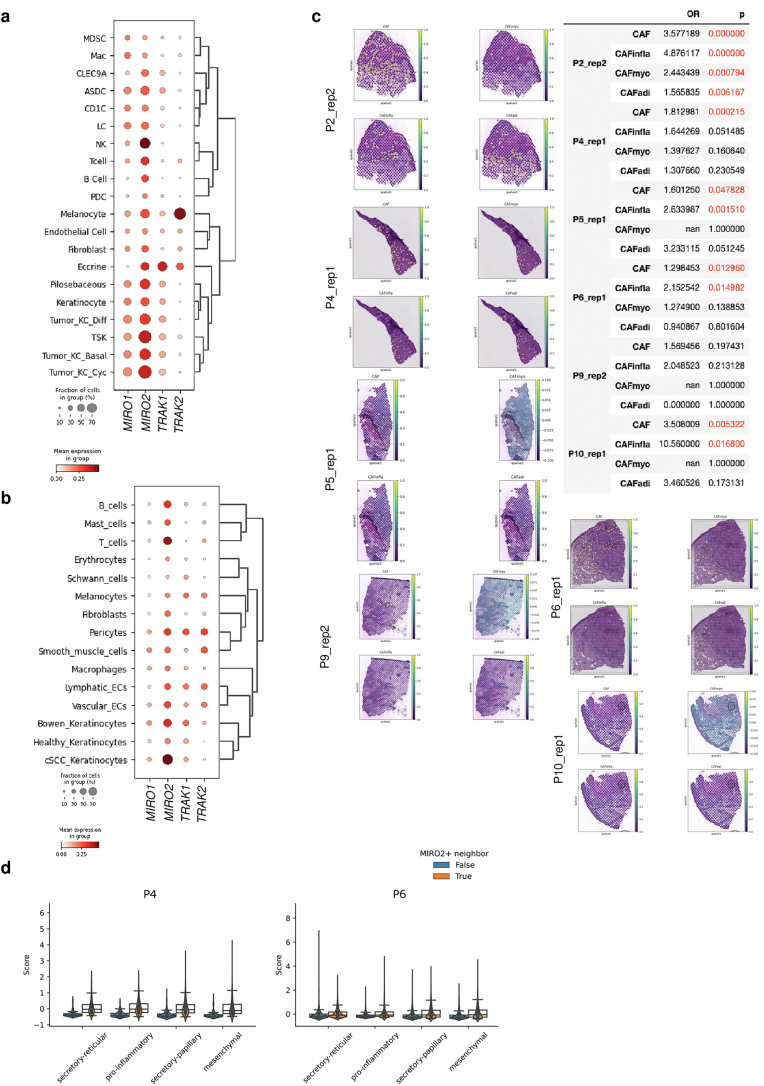
*MIRO2* is predominantly expressed in tumor keratinocytes of SCCs. **a**. Ridge plot showing abundance of mRNAs encoding mitochondrial trafficking proteins in different cell types based on published scRNA-seq data from human cutaneous SCCs^40^. *n* = 10 tumors and patient- and site-matched normal skin collected from 10 individuals. **b**. Ridge plot showing abundance of mRNAs encoding mitochondrial trafficking proteins in different cell types based on published scRNA-seq data from human cutaneous SCCs^28^. *n* = 5 invasive SCCs and *n* = 6 normal skin samples. **c**. Odds ratio analysis of MIRO2 transcript co-localization with CAF subtypes in patient tumor sections. The table correlates *MIRO2* expression with expression of *PDGFRA* and specific markers for CAF subtypes—*MMP11* for CAFinfla, *ACTA2* for CAFmyo, and *CFD* for CAFadi—quantified by odds ratios (OR) across patient samples. *n* = 6 tumor sections. Accompanying the table, patient tissue sections are illustrated, with transcript-positive spots for MIRO2 and CAF markers denoted by colored dots, showcasing the spatial interplay within the tumor microenvironment. OR values denote co-localization strength, and p-values indicate statistical significance. P2_rep2: *n* = 646, P4_rep1: *n* = 744, P5_rep1: *n* = 590, P9_rep2: *n* = 1,071, P6_rep1: *n* = 3,650, P10_rep1: *n* = 608. Significant values are in red. **d**. Comparison of CAF score distribution between MIRO2+ spots and their neighbors (True) and remaining spots (False). *n* = 2 tumors from different patients (referred to as P2 and P6 from Ji et al.^40^). Violin plots in (**d**) show the median (center line), 25^th^ and 75^th^ percentiles (box bounds), and the whiskers extend to the most extreme data points within 1.5 times the interquartile range from the box. Points outside this range are plotted as outliers. Fisher’s exact test was used to determine statistical significance (**c**). Source data

To identify potential regulators of the mitochondrial transfer from skin cancer cells to fibroblasts, we used published single-cell RNA (scRNA)-seq data of tumors and site-matched normal skin from persons with cutaneous SCCs^40^. We used preprocessed annotations to identify keratinocyte populations^40^ and focused on genes with a documented function in mitochondrial trafficking, including *MIRO1 (RHOT1)*, *MIRO2 (RHOT2)*, *TRAK1* and *TRAK2* (ref. ^41^). We defined a gene as ‘highly expressed’ when its expression level exceeded the mean expression level observed across all cell populations examined. Expression of *MIRO2* was significantly elevated in malignant versus nonmalignant epithelial cells (Fig. 6a). Tumor-specific keratinocytes (TSKs), a cluster exclusively present in tumor samples^40^, exhibited particularly high *MIRO2* expression (Fig. 6b). This was confirmed with another scRNA dataset from skin SCCs^28^ (Extended Data Fig. 7a,b). Analysis of spatial transcriptomics data revealed elevated *MIRO2* expression in invasively growing cells at tumor margins (Fig. 6c). To delineate MIRO2 mRNA localization relative to CAF subtypes, we used coexpression analysis with *PDGFRA* and specific markers for iCAFs (*MMP11*), myCAFs (*ACTA2*) and adipose CAF (adiCAF; *CFD*)^30^. We detected substantial colocalization of MIRO2 mRNA with these CAF subtypes, particularly with MMP11-positive iCAFs (Extended Data Fig. 7c). We further applied Cell2Location^42^ spatial deconvolution using skin SCC data as a single-cell (ref. ^28^) to estimate cell type distributions per spot. We computed CAF scores using fibroblast-specific expression inferred by Cell2Location and identified MIRO2^+^ spots on the basis of keratinocyte-specific expression. CAF scores were consistently higher in MIRO2^+^ spots and neighboring regions compared to non-MIRO2^+^ spots, although only minor differences were observed among the four CAF subtypes (Extended Data Fig. 7d).

Western blot analysis showed strong expression of MIRO2 in A431 and HaCaT-Ras cells but it was hardly detectable in the parental HaCaT cells and in fibroblasts (Fig. 6d). The predominant expression of MIRO2 in the epithelium was confirmed by immunostaining of three-dimensional (3D) organotypic cultures (Fig. 6e).

**Extended Data Fig. 8:**
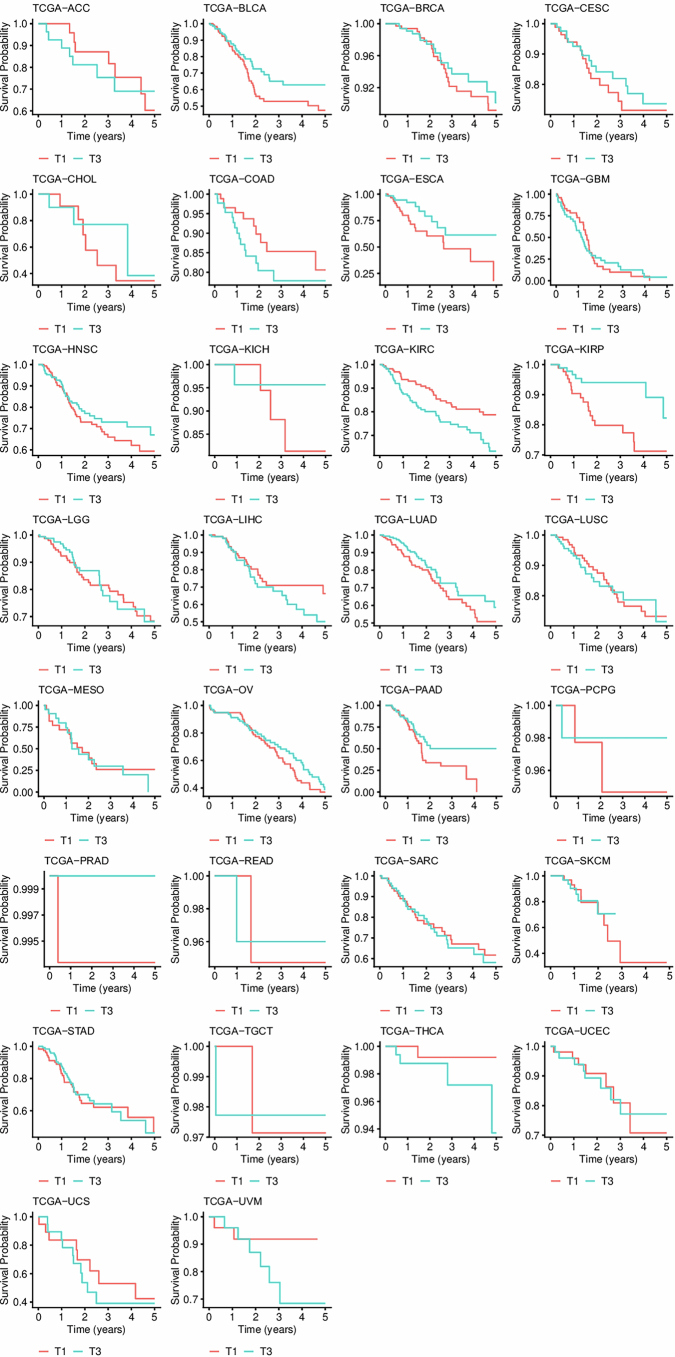
Global *MIRO2* expression does not correlate with survival across different human tumors. Kaplan-Meier plots showing the disease specific survival (DSS) rate by cancer type in TCGA patients with high or low expression of *MIRO2*. T1 (Tercile 1; lowest): low expression of *MIRO2*; T3 (Tercile 3; highest): high expression of *MIRO2*. Source data

Increased *MIRO2* expression did not consistently correlate with poor survival across 30 different tumors (Fig. 6f and Extended Data Fig. 8). However, the cancer expression data are based on bulk cancer tissue, whereas the expression of *MIRO2* was mainly upregulated at the tumor edge. Indeed, analysis of the leading edge signature from oral SCC^43^ showed a positive correlation of this signature with *MIRO2* expression across most of the 30 cancer types (Fig. 6g). This is of likely functional relevance, because Cancer Dependency Map (DepMap) analysis showed an important role of MIRO2 in the proliferation and survival of cancer cells (Fig. 6h).

### MIRO2 promotes mitochondrial transfer

**Fig. 7:**
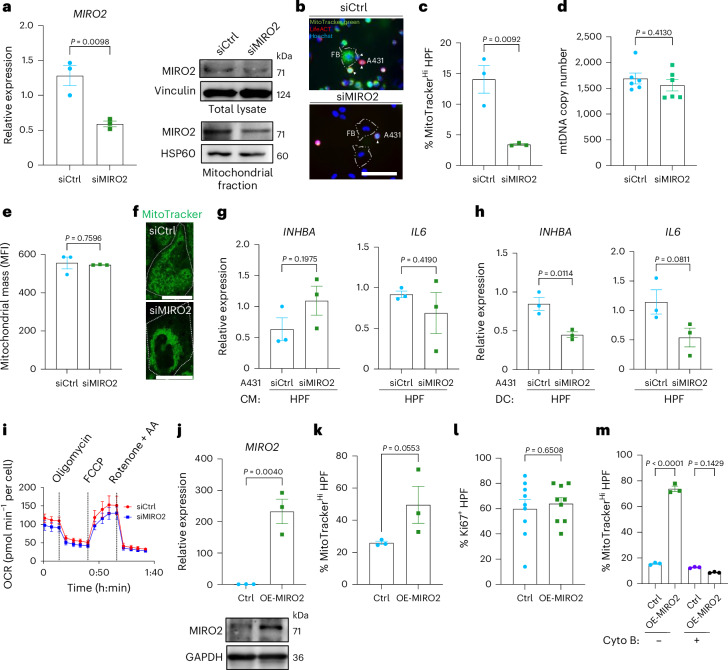
MIRO2 is required for mitochondrial transfer. **a**, RT–qPCR for *MIRO2* using RNA from siCtrl or siMIRO2 A431 cells; Western blot of total and mitochondrial lysates from these cells for MIRO2, vinculin or HSP60 (loading controls) (*n* = 3 cultures per group). **b**, Fluorescence images of LifeAct A431 cells (red) stained with MitoTracker green and transfected with siCtrl or siMIRO2 in coculture with HPFs, counterstained with Hoechst. White arrowheads indicate A431 cells. **c**, Percentage of MitoTracker-high HPFs in cocultures with siMIRO2 or siCtrl A431 cells, normalized to the number of cancer cells (*n* = 3 cocultures per group). **d**, qPCR for mtDNA encoding tRNA-Leu(UUR) relative to nucDNA encoding B2M using DNA from A431 cells transfected with siCtrl or siMIRO2. Total mtDNA content was calculated on the basis of *C*_*t*_ values (*n* = 3 cultures per group). **e**, Mitochondrial mass in siCtrl and siMIRO2 A431 cells based on MitoTracker green mean fluorescence intensity (MFI) (*n* = 3 per group). **f**, Confocal images of siCtrl or siMIRO2 A431 cells incubated with MitoTracker green. The dashed line marks the outer edge of the cell (*n* = 3 cultures per group). **g**, RT–qPCR for *INHBA and IL6* using RNA from HPFs incubated with CM of siCtrl or siMIRO2 A431 cells (*n* = 3 cultures per treatment group). **h**, RT–qPCR for *INHBA* and *IL6* using RNA from sorted HPFs cocultured with siCtrl or siMIRO2 A431 cells (*n* = 3 cocultures per group). DC, direct culture. **i**, OCR of siCtrl or siMIRO2 A431 cells. The time of drug injection is indicated (*n* = 3 cultures per group). **j**, RT–qPCR for *MIRO2* using RNA from A431 cells transfected with control or MIRO2 overexpression vectors (OE-MIRO2) (*n* = 3 cultures per group). Western blot of lysates from control or MIRO2-overexpressing A431 cells for MIRO2 or GAPDH (*n* = 2 cultures per group). **k**, Percentage of MitoTracker-high HPFs after coculture with MitoTracker-stained control or MIRO2-overexpressing A431 cells (*n* = 3 cocultures per group). **l**, Percentage of Ki67^+^ HPFs after coculture with control or MIRO2-overexpressing A431 cells (*n* = 9 cocultures per group). **m**, Percentage of MitoTracker-high HPFs after coculture with MitoTracker-stained control or MIRO2-overexpressing A431 cells, with or without treatment with dihydrocytochalasin B (*n* = 3 cocultures per group). Graphs show the mean ± s.e.m. An unpaired two-sided Student’s *t*-test (**a**,**c**–**e**,**g**,**h**,**l**) or two-sided one-way ANOVA with Bonferroni post hoc multiple comparison test (**m**) was used to determine statistical significance. Scale bars, 100 μm (**b**) and 25 μm (**f**). Source data

**Extended Data Fig. 9:**
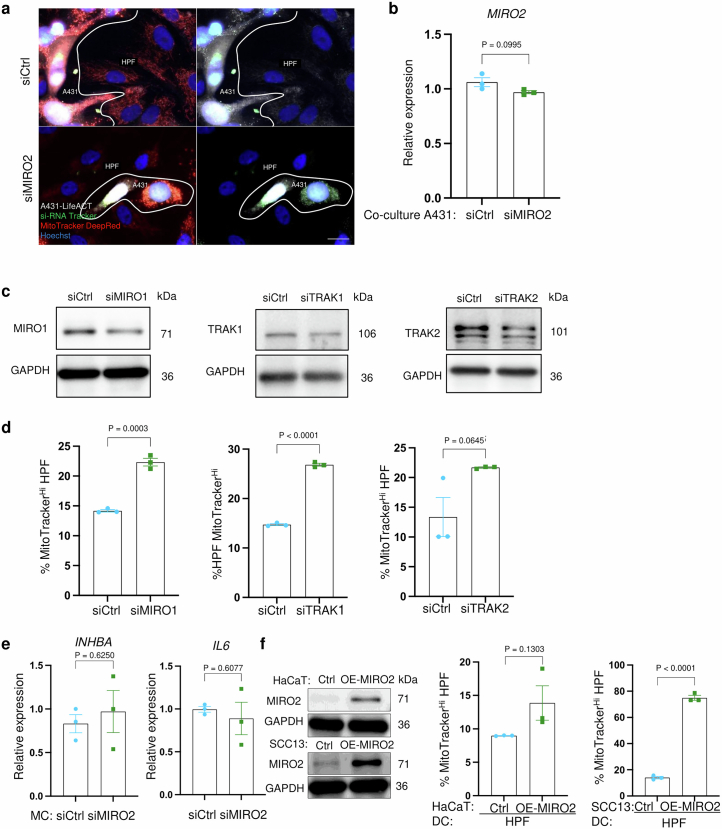
MIRO2 promotes mitochondrial transfer from cancer cells to HPFs via TNTs. **a**. Representative photomicrographs of co-cultures of LifeAct-A431 cells, pre-labeled with MitoTracker DeepRed (red) and transfected with siCtrl or siMIRO2 pre-labeled with Label IT® siRNA Tracker™ fluorescein (green), with HPFs. Cultures were counterstained with Hoechst (blue). Note the exclusive detection of the labeled siRNA in A431 cells. Scale bar: 25 μm. *n* = 3 co-cultures. **b**. qRT-PCR for *MIRO2* relative to *RPL27*, using RNA from HPFs sorted following co-culture with siCtrl or siMIRO2 A431 cells. *n* = 3 co-cultures per group. **c**. Western blot analysis of total lysates from A431 cells transfected with siCtrl, siMIRO1, siTRAK1, or siTRAK2 for MIRO1, TRAK1, TRAK2, and GAPDH (loading control). *n* = 2 cultures per group. **d**. Mitochondrial transfer efficiency between A431 cells transfected with siMIRO1, siTRAK1, siTRAK2, or siCtrl and HPFs in co-culture, based on the proportion of MitoTracker-high fibroblasts. *n* = 3 co-cultures per group. **e**. qRT-PCR for *INHBA* and *IL6* using RNA from HPFs subjected to MitoCeption with mitochondria from A431 cells, which had been transfected with siCtrl or siMIRO2. *n* = 3 transfected cultures per group. **f**. Western blot analysis for MIRO2 using lysates from HaCaT or SCC13 cells transfected with control or MIRO2 overexpression vectors (OE-MIRO2). *n* = 3 cultures per group. Bar graphs show percentage of MitoTracker-high HPFs after direct co-culture (DC) with control or MIRO2-overexpressing cells. *n* = 3 co-cultures per group. Graphs show mean ± SEM. Unpaired two-sided Student’s t-test was used to determine statistical significance (**b**, **d**–**f**). Source data

We next investigated the impact of MIRO2 knockdown on intercellular mitochondrial transfer by establishing cocultures of HPFs and LifeAct A431 cells, which were transfected with MIRO2 or scrambled siRNAs and stained with MitoTracker green (Fig. 7a,b). Transfection with fluorescently labeled siRNA showed no detectable transfer of siRNA to cocultured HPFs (Extended Data Fig. 9a). Consistently, HPFs isolated from cocultures of A431 cells transfected with siMIRO2 or siCtrl showed no significant difference in *MIRO2* expression (Extended Data Fig. 9b).

Mitochondrial transfer was significantly reduced when HPFs were cocultured with siMIRO2 versus control A431 cells (Fig. 7c), while knockdown of MIRO1, TRAK1 and TRAK2 even increased the transfer (Extended Data Fig. 9c,d). MIRO2 knockdown did not lead to a decrease in mitochondrial DNA copy number or mitochondrial mass (Fig. 7d,e) and did not impede the activation of CAF marker expression through MitoCeption (Extended Data Fig. 9e), suggesting that MIRO2 is mainly responsible for the mitochondrial transfer rather than the effect on the recipient fibroblasts.

The impaired mitochondrial transfer by MIRO2-knockdown cells correlated with perinuclear clustering of mitochondria (Fig. 7f). This is relevant, because proper distribution of mitochondria is important for protein secretion and cell migration^16,44^. It is consistent with the role of MIRO family proteins in the regulation of mitochondrial distribution^45^. The depletion of mitochondria at the periphery of cancer cells is likely to impact the mitochondrial transfer to fibroblasts. Incubation of fibroblasts with the CM of siMIRO2 versus control A431 cells did not significantly affect CAF marker gene expression, in contrast to the effect of MIRO2 knockdown in direct coculture (Fig. 7g,h). These findings again demonstrate that direct contact between cancer cells and fibroblasts, which allows mitochondrial transfer through TNTs, is necessary for the induction of a CAF phenotype.

Depletion of MIRO2 did not significantly impact the metabolic activity of A431 cells (Fig. 7i). Therefore, the perinuclear clustering of mitochondria upon MIRO2 depletion is not attributed to major deficiencies in mitochondrial respiration or metabolism. Instead, it is likely a consequence of altered mitochondrial motility and distribution within the cell.

Overexpression of MIRO2 promoted transfer activity of A431 and SCC13 cells and a mild effect was also seen for HaCaT cells, as shown by flow cytometry analysis of MitoTracker-high HPFs (Fig. 7j,k and Extended Data Fig. 9f). However, the increase in mitochondrial transfer from A431 cells to HPFs did not further promote HPF proliferation (Fig. 7l). Together with the results from MitoCeption studies with different amounts of mitochondria (Fig. 4k), these data suggest a threshold for CAF reprogramming, beyond which further mitochondrial transfer to fibroblasts has no additional effect. Inhibition of actin polymerization nearly abolished the elevated mitochondrial transfer from MIRO2-overexpressing A431 cells to fibroblasts (Fig. 7m). The selective effect of MIRO2 knockdown or overexpression on the number of MitoTracker-positive fibroblasts further confirms the suitability of MitoTracker staining under our experimental conditions, as dye leakage would not be affected by these treatments.

### MIRO2 regulates skin tumorigenesis in mouse models

**Fig. 8:**
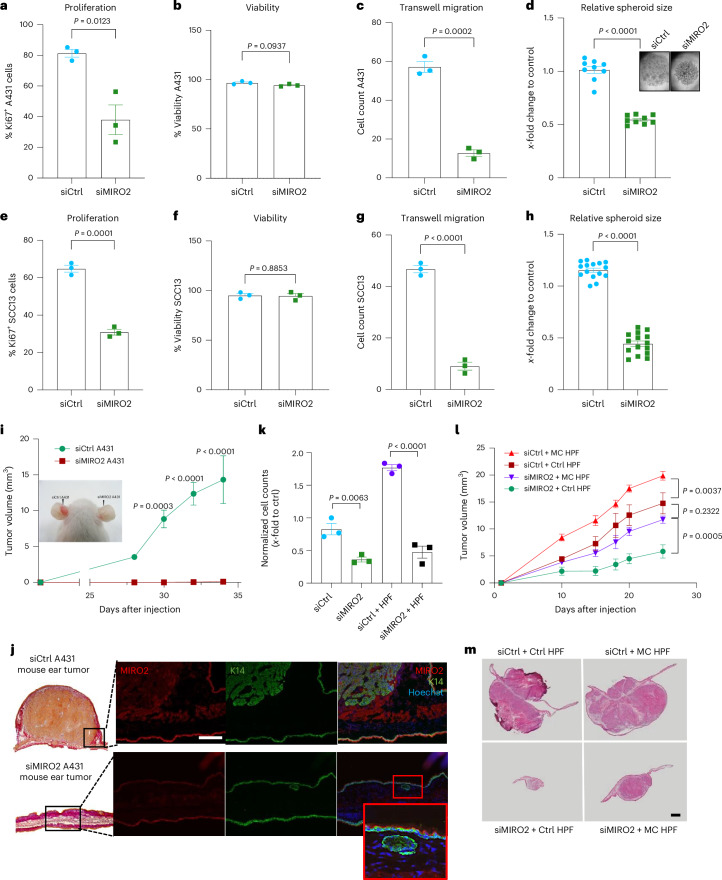
MIRO2 depletion in cancer cells reduces mitochondrial transfer and tumor growth. **a**, Percentage of Ki67^+^ A431 cells 24 h after transfection with siCtrl or siMIRO2 (*n* = 3 cultures per group). **b**, Relative viability of A431 cells 24 h after transfection with siCtrl or siMIRO2 (*n* = 3 cultures per group). **c**, Transwell migration of A431 cells 24 h after transfection with siCtrl or siMIRO2 (*n* = 3 cultures per group). **d**, Relative spheroid area of a single hanging drop formed by siCtrl or siMIRO2 A431 cells and representative images of the spheroids (*n* = 9 spheroids per group). **e**, Percentage of Ki67^+^ SCC13 cells 24 h after transfection with siCtrl or siMIRO2 (*n* = 3 cultures per group). **f**, Relative viability of SCC13 cells 24 h after transfection with siCtrl or siMIRO2 (*n* = 3 cultures per group). **g**, Transwell migration of SCC13 cells 24 h after transfection with siCtrl or siMIRO2 (*n* = 3 cultures per group). **h**, Relative spheroid area of a single hanging drop formed by siCtrl or siMIRO2 SCC13 cells and representative images of the spheroids (*n* = 15 spheroids per group). **i**, Photo of 5-week-old ear xenograft tumors (indicated by arrowheads) formed after injection of 200,000 A431 cells transfected with siMIRO2 or siCtrl and tumor volume at different time points (*n* = 3 tumors per group from different mice). **j**, Representative photomicrographs of Herovici-stained tumors (left) formed by siMIRO2 or siCtrl A431 cells and immunofluorescence staining of sections from these tumors for K14 (green) and MIRO2 (red), counterstained with Hoechst (blue). Inset, a tumor cell island with persistent MIRO2 knockdown (*n* = 3 sections from independent tumors per group). **k**, Normalized cell count of A431 LifeAct–RFP cells transfected with siCtrl or siMIRO2, cocultured with or without HPFs in spheroids and analyzed by FACS after 5 days (*n* = 3 spheroids per group). **l**, Tumor volume during tumor development following coinjection of HPFs (with or without A431-derived mitochondria, introduced using MitoCeption) and A431 cells transfected with either siMIRO2 or siCtrl (*n* = 5 tumors per group). **m**, Representative H&E stainings of tumors from each group (*n* = 5 mice). Graphs show the mean ± s.e.m. An unpaired two-sided Student’s *t*-test (**a**–**h**) or two-sided one-way (**k**) or two-way (**i**,**l**) ANOVA with Bonferroni post hoc multiple comparison test (**i**,**k**,**l**) was used to determine statistical significance. Scale bars, 100 μm (**d**), 200 μm (**j**) and 1 mm (**m**). Source data

Next, we tested the impact of MIRO2 depletion on the malignant features of A431 cells. A 24-h knockdown of MIRO2 significantly reduced their proliferation without impacting their viability (Fig. 8a,b). MIRO2-knockdown cells also migrated more slowly than control cells in a transwell assay and formed smaller, less developed 3D spheroids (Fig. 8c,d). Similar results were obtained with SCC13 cells (Fig. 8e–h). Lastly, NOD scid mice injected with siMIRO2 A431 cells failed to develop tumors, whereas control cells rapidly formed large tumors (Fig. 8i,j). Microscopic examination of the ear tissue from the siMIRO2 A431 group revealed small, undeveloped cancer cell colonies with undetectable MIRO2 expression (Fig. 8j, inset). Therefore, even a transient reduction of MIRO2 levels during the early phase of tumor formation is sufficient to prevent tumorigenesis.

To determine whether the poor spheroid growth of MIRO2-knockdown cells and their failure to form tumors in mice is simply a consequence of their cell-autonomous defect in proliferation or migration or whether it involves non-cell-autonomous effects, such as impaired mitochondrial transfer to fibroblasts, we set up coculture spheroid assays. The difference in proliferation between siCtrl and siMIRO2 cancer cells was even more pronounced in the presence of fibroblasts (Fig. 8k), indicating an important non-cell-autonomous role of MIRO2. In xenograft experiments, coinjection of siMIRO2 A431 cells with control HPFs already caused a mild stimulation of tumor growth but the tumor-promoting effect was much stronger with MitoCepted HPFs. This combination compensated for the deficiency of siMIRO2 cancer cells in tumor formation and the rate of tumor growth was almost comparable to that of the control group, in which siCtrl A431 cells were coinjected with control fibroblasts (Fig. 8l,m). These findings highlight the notable influence of fibroblasts with mitochondria from cancer cells on tumor formation and further suggest an important role of MIRO2 in this transfer.

## Discussion

We identified mitochondrial transfer through TNTs as a strategy of cancer cells to promote CAF differentiation. Because cancer cells and CAFs often have direct contact in the tumor, particularly at its periphery^46^, this transfer is likely to contribute to the increased invasiveness of cancer cells. We further show that CAF differentiation through mitochondrial transfer from cancer cells is supported by two mechanisms. First, cancer cells at the invasive front overexpress *MIRO2*, which promotes the mitochondrial transfer. Second, mitochondria from malignant cells but not from nontumorigenic epithelial cells can induce a CAF phenotype. This could be explained by alterations in the proteome of mitochondria from cancer cells^47,48^ and the associated metabolic alterations^49^. In support of this hypothesis, uptake of mitochondria from A431 cancer cells altered the expression of several metabolic proteins and promoted OxPhos and ATP production in the recipient fibroblasts. These features were shown to promote proliferation, matrix production and protein secretion by fibroblasts and CAFs^24,37,50^. Consistent with an important role of OxPhos in the fibroblast reprogramming by mitochondrial transfer, inhibition of this metabolic pathway in the recipient fibroblasts blocked the induction of important CAF features. Therefore, metabolic alterations induced by mitochondria from cancer cells contribute to the CAF phenotype.

The induction of a CAF phenotype was associated with significant changes in the expression of genes and proteins associated with inflammation, immune response, cellular metabolism and stress response. In the cocultures, we found increased expression of many ISGs. This is consistent with the sensing of genomic damage of cancer cells by fibroblasts, which resulted from transcytosis of cytoplasm from cancer cells into neighboring fibroblasts and activation of the stimulator of interferon genes–interferon regulatory factor 3 pathway^51^. However, ISG expression was not upregulated in the MitoCepted fibroblasts, demonstrating that they are not notably affected by cancer cell mitochondria. By contrast, several classical CAF markers were upregulated in the MitoTracker-high population and in MitoCepted fibroblasts, suggesting that coculture with cancer cells alters the expression of different sets of genes in fibroblasts through distinct mechanisms.

In a search for the mechanistic underpinning of the mitochondrial transfer, we identified MIRO2. This Rho guanosine triphosphatase links mitochondria to the cellular trafficking machinery and is responsible for intracellular mitochondrial positioning^52^. It also has important cell-autonomous functions that are important for cancer cell proliferation and migration^53^, as also shown in this study. These features and its overexpression in invasive cancer cells at the edge of different tumors make it an interesting target for cancer treatment. This is not restricted to skin cancer because we also observed mitochondrial transfer from vulvar, breast and pancreatic cancer cells to fibroblasts, which promoted a CAF phenotype. Given the high expression of MIRO2 in metastatic prostate cancer^53^, it will be of interest to study the role of intercellular mitochondrial transfer in metastasis.

In conclusion, we show that cancer cells transfer their mitochondria to fibroblasts and thereby reprogram them into protumorigenic CAFs. We also discovered the mechanism underlying this transfer and identified MIRO2 as a potential target for cancer treatment. These findings offer promising therapeutic opportunities for skin cancer and also for malignancies with high mortality, such as pancreatic cancer, which has a large stromal component^54^. Lastly, the data obtained in this study suggest that mitochondrial transfer from epithelial to stromal cells is an important mechanism of cell–cell communication, which may also be relevant for development, homeostasis and tissue repair and for the pathogenesis of nonmalignant diseases.

## Methods

### Ethics statement

The work performed in this study complies with all relevant ethical regulations.

Mouse maintenance and experimentation were approved by the veterinary authorities of Zurich (Kantonales Veterinäramt Zurich, 32060, 35555, 36338 and 33866).

Human skin and tumor samples, which were used for the isolation of primary cells, were obtained anonymously from the Department of Dermatology, University Hospital of Zurich (in the context of the biobank project). Written informed consent for use in research was obtained from all donors (in case of foreskin from the parents).

All experiments with human samples were approved by the local and cantonal Research Ethics Committees (Kantonale Ethikkommission Zurich, BASEC no. 2017-00684), adhering to the Declaration of Helsinki Principles.

### Mouse maintenance

NOD scid (NOD.CB17-*Prkdc*^*scid*^/NCrCrl) mice were bred in the ETH Zurich EPIC facility and kept under specific pathogen-free conditions in a 12 h dark–light cycle at 21–23 °C and 40–60% humidity. They received food and water ad libitum.

### Cell lines

HaCaT, HaCaT-Ras cells and SCC13 cells were provided by P. Boukamp. A431 cells were from Merck (85090402). LifeACT cell lines were generated by transduction with the lentiviral vector rLV-Ubi-LifeAct–RFP-Tag (Vitaris). HaCaT cells are spontaneously immortalized but nontumorigenic human keratinocytes^21^. HaCaT-Ras cells were obtained by transfection of HaCaT cells with a *c-HA–RAS* oncogene^32^. SCC13 cells were derived from a human cutaneous SCC^31^. The metastatic MDA-MB-231 triple-negative breast cancer cell line and the metastatic PANC1 pancreatic cancer cell line were obtained from the American Type Culture Collection (HTB-26, CRL-1469). LM2 cells, a lung metastatic variant of MDA-MB-231, were kindly provided by J. Massagué. Cell lines expressing fluorescent mitochondrial proteins were generated by lentiviral transduction^35,55^. Primary fibroblasts expressing TOM20–GFP were generated by lentiviral transduction with pLenti-X1-blast-GFP-TOM20-MTS, provided by J. Corn (ETH Zurich). Immortalized mouse fibroblasts were isolated from PDGFRα–eGFP transgenic mice and spontaneously immortalized by serial passaging^56^. Authentication of HaCaT, HaCaT-Ras, SCC13 and A431 cells was performed by Microsynth using highly polymorphic short tandem repeat loci (most recently in February 2025). Absence of *Mycoplasma* was confirmed monthly using the PCR *Mycoplasma* test kit I/C (PromoKine) or the MycoStrip kit (InvivoGen). Cells were cultured in DMEM supplemented with 10% FBS and 1% penicillin–streptomycin (complete DMEM), unless indicated otherwise.

### Human primary cells

HPKs from skin of adult healthy volunteers or from the edges of skin SCCs of adult participants (diagnosed by an experienced dermatopathologist) were from H.-D. Beer (University Hospital Zurich). Foreskin HPFs were obtained from foreskin of healthy boys.

Human skin and tumor samples, which were used for the isolation of primary cells, were obtained anonymously from the Department of Dermatology, University Hospital of Zurich (in the context of the biobank project). Written informed consent for use in research was obtained from all donors (in case of foreskin from the parents).

All experiments with human samples were approved by the local and cantonal Research Ethics Committees (Kantonale Ethikkommission Zurich, BASEC no. 2017-00684), adhering to the Declaration of Helsinki principles.

HPKs were cultured in keratinocyte serum-free medium with epidermal growth factor and bovine pituitary extract (all from Thermo Fisher Scientific). HPFs were cultured in complete DMEM.

### siRNA-mediated knockdown

Cancer cells were transfected with siRNAs (Microsynth AG) using Lipofectamine RNAiMAX (Thermo Fisher Scientific) and incubated for 24–72 h or retransfected after 72 h and incubated for an additional 24 h. The following siRNAs were used:

Connexin 26 siRNA (72 h): 5′-CCCAGUUGUUAGAUUAAGATT-3′

MIRO1 siRNA (72 h + 24 h): 5′-UAACCAAAUCGUCGAAGCACAGUCCTT-3′

MIRO2 siRNA (24 h): 5′-GCGUGGAGUGUUCGGCCAATT-3′

SEC3 siRNA (72 h): 5′-AGAUGAAUACCAAGAGUUA-dTdT-3′

SEC5 siRNA (72 h): 5′-GGGUGAUUAUGAUGUGGUUdTdT-3′

TRAK1 siRNA (72 h): 5′-GGAAACGAUGAGCGGAGUATT-3′

TRAK2 siRNA (72 h): 5′-GGAUAGAUAUGCACUGAAATT-3′

Negative control siRNA: 5′-AGGUAGUGUAAUCGCCUUG-3′

### MIRO2 overexpression

First, 1 μg of pRK5-myc-MIRO2 expression vector (Addgene, 47891) or empty vector were used for transfection using Lipofectamine 2000 (11668019, Thermo Fisher Scientific). DNA, Lipofectamine and Opti-MEM reduced-serum medium (31985062, Thermo Fisher Scientific) were incubated at room temperature for 20 min. Cells were incubated with the mixture for 6 h, followed by incubation in complete DMEM for 24 h.

### In vivo tumorigenesis assays

Xenograft skin tumorigenesis assays were performed as described^24^ by intradermal injection of 2 × 10^5^ cancer cells or 10^5^ cancer cells together with 10^5^ fibroblasts into the ear of male NOD scid mice at the age of 8–12 weeks.

Breast and pancreatic cancer xenografts were established by orthotopic injection of LM2 breast cancer cells into the mammary fat pad (glands 2–3) of adult female NOD scid mice^57^ or by direct injection of PANC1 cells into the pancreas of adult mice.

The maximal tumor size permitted by the ethics committee (1-cm diameter for skin cancer, 2.8-cm^2^ volume for breast cancer) or the end point for wellbeing (hunching, piloerection or decreased activity for pancreatic cancer) was never reached in our experiments.

### Spheroid formation

Spheroid assays were performed using the experimental parameters proposed by The MISpheroID Consortium^58^. A total of 2,000 cancer cells in 20 μl of CM from HPFs, which were cultured in complete DMEM, were placed on the lids of 6-cm culture plates using a hanging-drop method^24^. Then, 5 ml of PBS was added to the bottom.

### Coculture of fibroblasts with epithelial cells

Equal numbers of HaCaT or cancer cells and fibroblasts were seeded to reach 80–100% confluency. Before coculture, cells were stained with MitoTracker or PKH67 cell linker (PKH67GL, Sigma-Aldrich) and washed with PBS. For imaging, cells were cultured on glass coverslips in complete DMEM and imaged using an Axio Imager M2 microscope equipped with an Axiocam MR camera and ZEN 2 software or using an Axioscan 7 microscope slide scanner equipped with a color Axiocam 705 color complementary metal–oxide–semiconductor camera and a fluorescence Axiocam 712 mono camera (all from Carl Zeiss). Image processing and analysis were performed using Fiji ImageJ (https://imagej.net/Fiji) or QuPath^59^.

For mechanistic studies, cocultures were treated with dihydrocytochalasin B (100 nM), nocodazole (10 μM) or carbenoxolone (100 μM) (all from Sigma-Aldrich) for 24 h.

### FluidFM

Injection of mitochondria was performed using a FluidFM setup as previously described^35^. Imaging was carried out using a spinning disc confocal microscope (Visitron Systems) with a Yokogawa CSUW1 scan head and an electron-multiplying charge-coupled device camera system (Andor Oxford Instruments). A total of 100,000 fibroblasts were seeded in two-well microinsets 6 h before injection. The microfluidic probe was positioned over individual fibroblasts and mitochondria were inserted using a cantilever system. Z stacks were taken to identify successful transfer of mitochondria. Consequently, 100 fibroblasts were injected and fixed in 4% paraformaldehyde (PFA) for 24 h after transfer and analyzed by immunofluorescence staining.

### Mitochondrial extraction

Mitochondria were extracted from 20,000,000 epithelial cells using a mitochondria isolation kit (89874, Thermo Fisher Scientific).

### MitoCeption

MitoCeption was performed as described^33^ using preseeded cells in six-well culture plates. A total of 100,000 HPFs were seeded the day before MitoCeption. Mitochondria isolated from 100,000 cells of various cell lines or primary cells, which included similar amounts of total protein (Extended Data Figs. 4c and 6d), were added to the bottom, ensuring even distribution. Culture plates were centrifuged at 1,500*g* for 15 min at 4 °C and incubated at 37 °C and 5% CO_2_ for 2 h. The centrifugation procedure was repeated and cells were cultured for 24 h before further processing.

For OxPhos inhibition, recipient fibroblasts were treated with 1 μM oligomycin (O4876, Sigma-Aldrich) for 24 h.

All experiments were performed with consistent exposure time of cells to mitochondria, uniform mitochondrial uptake across experiments and standard post-transplantation conditions. Data were normalized to the number of donor cells.

### FACS and flow cytometry

Cells were stained with Sytox Blue (S34857, Thermo Fisher Scientific) before acquisition or analysis. Live MitoTracker-high and MitoTracker-low cells or Su9–RFP-high and Su9–RFP-low cells were sorted using a FACSAria Fusion sorter (BD Biosciences) with a 100-μm nozzle and 20 psi pressure on the basis of their fluorescence intensity. The number of cells was normalized after each FACS run. To analyze proliferation, cells were fixed and permeabilized using a Foxp3 transcription factor staining buffer set (00-5523-00, eBioscience) before intracellular staining with PE-conjugated (sc-7846, Santa Cruz) or PE–Cyanine7-conjugated (25-5698-82, eBioscience) anti-Ki67 for 1 h at room temperature.

For mitochondrial content analysis, cells were stained with MitoTracker green FM (M46750, Thermo Fisher Scientific).

Flow cytometry was performed using an LSR Fortessa or a FACSAria Fusion cell analyzer (both from BD Biosciences). Data were analyzed using FlowJo version 10.10 software (BD Biosciences).

### Transwell migration assays

Chemotactic transwell migration was assessed as described previously^24^ and cancer cells were allowed to migrate for 24 h toward CM from fibroblasts.

### Analysis of mitochondrial transfer in transwell cocultures

Transwell coculture assays were previously described^18^.

### Seahorse XF cell mito stress test

A total of 100,000 cells per well were seeded on XF96 Seahorse plates in full medium. The medium was then switched to Seahorse XF base medium (103335-100, Agilent Technologies) supplemented with 10 mmol L^−1^ glucose, 1 mmol L^−1^ sodium pyruvate and 2 mmol L^−1^ glutamine (assay concentration, https://www.agilent.com/cs/library/usermanuals/public/XF_Cell_Mito_Stress_Test_Kit_User_Guide.pdf) and incubated in a CO_2_-free incubator for 1 h. Oligomycin, carbonyl cyanide-*p*-trifluoromethoxyphenylhydrazone (FCCP) and antimycin A (AA) + rotenone were prepared in the XF assay medium with final concentrations of 1, 1.5 and 1/0.1 μmol L^−1^, respectively, and provided by the Seahorse XF cell mito stress test kit (103015-100; Agilent Technologies). The compounds were injected to assess the OCR of cells in an XF96 plate. Metabolic flux data were normalized to cell count, which was determined using Hoechst staining and analysis in a fluorescence reader (Agilent Technologies, BioTek Cytation 1) on the day of analysis.

### Measurement of ATP levels

ATP levels were determined using the CellTiter-Glo assay kit (Promega).

### Measurement of mitochondrial ROS

Mitochondrial ROS were measured as previously described^16^ using MitoSOX-Red (M36008, Thermo Fisher Scientific).

### ECM decellularization

ECM was decellularized with 0.5% Triton X-100 in 20 mM NH_4_OH for 1–3 min and washed with PBS before fixation with 4% PFA for 15 min at room temperature. Immunostaining was performed to analyze the expression of ECM proteins in the dECM. Hoechst staining was performed to assess the efficiency of decellularization.

### Histology, immunostaining and image analysis

Histological analyses and immunostainings were performed as described previously^24^ using the antibodies listed in Supplementary Table 2. Immunofluorescence images were analyzed using Fiji and staining intensity was normalized to cell number with at least nine microscopic fields of view for each condition analyzed. Mitochondrial networks were analyzed with MiNA as previously described^16^. The relative distance is indicated with values from 1 to 10; at least 100–200 intensity profiles were measured. Colony area and dECM area were measured after thresholding the images using ImageJ (Fiji). Colocalization analysis was adapted from Delaunay et al.^60^. Fluorescent intensity profile on the specified line was measured using ImageJ (Fiji) and normalized by the highest intensity value. All images from the same experiment were processed in an identical way by adjusting brightness and contrast and subtracting background signal to identify cell edge and contour or thresholding with the same values using a wide-field microscope.

The length of TNTs between cancer cells and fibroblasts was measured using the line or Polygon tool in QuPath.

### Holotomographic real-time imaging

Holotomographic imaging was performed with a Tomocube HT-X1 microscope (Tomocube). Cells were seeded on glass-bottomed dishes (P06-1.5H-N, Cellvis) at a density of 1 × 10^5^ cells per dish. The laser module was aligned for optimal illumination of the sample. The imaging process captured the cells’ refractive index and immunofluorescence labeling. Videos were acquired using a high-speed camera for 6 h with a time interval of 5 min. Finally, Tomocube software was used to process the acquired images.

### RNA isolation and RT–qPCR

RNA isolation and RT–qPCR were performed as described previously^24^ using the primers listed in Supplementary Table 3. Values obtained for the first control were set to 1.

### Western blot analysis

Western blot analysis was performed using standard procedures^24^ and antibodies to MIRO2 (H00089941-B01P, Novus Biologicals; 1:1,000 diluted), MIRO1 (NBP1-59021, Novus Biologicals; 1:500 diluted), TRAK1 (PA5-70029, Invitrogen; 1:500 diluted), TRAK2 (PA5-21858, Invitrogen; 1:500 diluted), EXOC1 (ab251853, Abcam; 1:500 diluted), EXOC2 (ab140620, Abcam; 1:500 diluted), vinculin (V4505, Sigma-Aldrich; 1:2,000 diluted), GAPDH (5G4, Hytest; 1:10,000 diluted) and HSP60 (Ab59457, Abcam, 1:500 diluted). Secondary antibodies were anti-rabbit or anti-mouse IgG (W4011 and W4021, Promega; 1:10,000 diluted) conjugated with horseradish peroxidase. Band intensities were quantified with ImageJ.

### DNA extraction for mtDNA quantification

Cells were collected with a cell scraper, transferred into Eppendorf tubes, and centrifuged at 6,500 rpm for 5 min. The pellet was resuspended in 200 µl of lysis buffer supplemented with 10 µl of proteinase K (10 mg ml^−1^; AppliChem). Samples were incubated overnight at 55 °C and then for 10 min at 95 °C to inactivate proteinase K and centrifuged at 17,000*g* for 10 min at room temperature. The supernatant, containing crude DNA, was retained for qPCR analysis.

### Mitochondrial genotyping

DNA was isolated from approximately 3 × 10^6^ immortalized fibroblasts from PDGFRα–eGFP transgenic mice and from A431 cells using a QIAamp DNA mini kit (51306, Qiagen). Both cell types were cocultured for 24 h, followed by FACS isolation of mouse fibroblasts. DNA was isolated and analyzed by PCR using primers that amplify the gene encoding human mitochondrial 16S RNA^10^. PCR products were visualized on a 2% agarose gel. The PCR product was purified using a QIAquick PCR purification kit (28104, Qiagen) and sequenced^10^. Species-specific and cell-type-specific SNPs were determined by comparing individual chromatograms using SnapGene software (GSL Biotech).

### Analysis of scRNA-seq and spatial transcriptomics data

scRNA-seq and spatial transcriptomics 10X Visium analysis was performed on the basis of published SCC datasets^28,40^. For scRNA-seq, prefiltered data were used according to the quality control procedures of each paper. The logged count per 10k (CP10k) was used for normalization. The distribution of expression of *MIRO2* was compared using a Wilcoxon rank-sum test. For spatial transcriptomics, the spots were normalized using the logged CP10k expression. To analyze the colocalization of MIRO2 mRNA positive spots with CAF subtypes, we first assigned spots to fibroblasts or CAFs (positive for *PDGFRA*), to an iCAF subtype (positive for *PDGFRA* and *MMP11*), to a myofibroblast CAF subtype (positive for *PDGFR*A and *ACTA2*), to an adiCAF subtype (positive for *PDGFRA* and *CFD*) or to *INHBA+* fibroblasts (expression of *INHBA* detected). To assess statistical significance of MIRO2^+^ and CAF subtype spots (INHBA^+^), we computed the six nearest neighbors of each spot and compared, using Fisher’s exact test, the enrichment of neighboring spots expressing both *MIRO2* and the CAF subtype marker or *INHB*A versus noncolocalized spots. For spatial transcriptomics data, the leading edge annotations were obtained from the authors.

### DepMap analysis

The effect of MIRO2 knockdown in genome-wide knockdown screens was analyzed using DepMap (DepMap.org).

### The Cancer Genome Atlas (TCGA) survival and gene signature analysis

TCGA clinical, survival and RNA-seq data from primary tumors of 8,911 participants across 30 solid cancer types were downloaded from the UCSC Xena data hub (https://xena.ucsc.edu)^61^ using the UCSCXenaTools R package (version 1.4.8)^62^. Gene expression values were downloaded as log_2_-transformed RSEM^63^ normalized counts.

The continuous *MIRO*2 gene expression was used for survival analysis censored at 5 years of follow-up. Hazard ratios were computed using the Cox proportional hazard model implemented in the ‘coxph’ function from the R package survival (version 3.5-7). For visualization, gene expression was divided into terciles and Kaplan–Meier survival curves were computed using the R package ggsurvfit (version 0.3.1).

The leading edge expression signature was composed by 91 genes upregulated at the leading edge compared to the tumor core^43^. For each TCGA sample, a signature enrichment score was computed using the ‘gsva’ method from the gene set variation analysis^64^ R Bioconductor package (version 1.46.0). The correlation between the leading edge signature score and *MIRO2* expression was assessed using Pearson’s correlation.

Random-effects meta-analysis across all cancer types was conducted with the ‘metagen’ function from the R package meta (version 7.0-0).

### RNA-seq and data analysis

Total RNA was isolated as described above. RNA-seq was performed after poly(A) enrichment and True-Seq library preparation on a Novaseq 6000 sequencer (Illumina).

#### Quantification of transcriptomic data and statistical analyses

FastQ files were trimmed using Trimmomatic^65^ (version 0.36), and processed using Salmon^66^ (version 1.10.2) using default parameters. The count matrix was processed using tximport^67^ to obtain gene-level counts and transcripts per million (TPM) estimates. PCA was computed on the standardized TPM expression. Differential expression among control, MitoTracker-high and MitoTracker-low fibroblasts was computed using PyDESeq2 (https://github.com/owkin/PyDESeq2), a Python variant of DESeq2 (ref. ^68^). Significance levels were cut at 10^−300^. To compare the markers of MitoTracker-high fibroblasts with known CAF subtypes, we defined a gene signature for MitoTracker-high fibroblasts using genes that were significantly differentially expressed compared to both MitoTracker-low fibroblasts (adjusted *P* < 0.01) and control populations (adjusted *P* < 0.01) and overexpressed in the MitoTracker-high population (fold change (FC) > 1.25). We computed the enrichment of this signature in known CAF subtype signatures with Fisher’s exact test, using genes quantified in the RNA-seq experiment as background.

#### Cell2Location spatial deconvolution

Single-cell data from human SCCs^28^ were used as a reference to deconvolve Visium 10X spots. A negative binomial model was trained on the discovery cohort with default parameters to estimate cell-type-specific average gene expression profiles. Next, the Cell2Location model was applied, setting the prior to *n* = 15 average cells per spot and α = 20 for relaxed regularization, which produced estimated counts of each cell type per spot. We next sampled from the model’s posterior distribution to determine cell-type-specific gene expression per spot. Fibroblast-specific expression estimates per spot were used to score CAF subtypes, leveraging marker genes from human SCCs^28^. Additionally, we assessed *MIRO2* expression using the estimated raw counts for keratinocytes.

### Proteomics and data analysis

#### Mass spectrometry (MS) sample preparation

Samples were prepared using SP3 technology^69^. Briefly, cell pellets were lysed in radioimmunoprecipitation assay buffer (50 mM Tris, 150 mM NaCl, 1% Triton X-100, 0.5% sodium deoxycholate and 0.1% SDS) and lysates were sonicated in an ultrasonic bath. Protein amount was quantified with a bicinchoninic acid assay (PIER23225, Pierce Biotechnology, Thermo Fisher Scientific). Then, 10 µg of protein was used for downstream analyses. After reduction of proteins with 5 mM dithiothreitol (DTT), they were alkylated with 5.5 mM iodoacetamide and quenched with 5 mM DTT. SP3 beads (Sera-Mag SpeedBeads, GE Healthcare, 45152105050250 and 65152105050250) were added to protein lysates in a 10:1 ratio. Binding to the beads was induced by the addition of 100% ethanol. After rinsing of beads with 80% ethanol, samples were digested overnight with trypsin (1:25) in 100 mM ammonium bicarbonate, pH 8. Peptides were desalted using STAGE tips and adjusted to a concentration of 100 ng µl^−1^ in 0.1% formic acid.

#### Liquid chromatography (LC)–MS/MS analyses

Peptides were analyzed by LC–MS/MS on a Vanquish Neo ultrahigh-performance LC instrument coupled to an Orbitrap Exploris 480 (both from Thermo Fisher Scientific) as previously described^70^. Briefly, samples were applied to fused silica C18 column tips (inner diameter: 75 μm; New Objective), produced in house with ReproSil-Pur 120 C18 AQ (1.9 μm, length: 20 cm; Dr. Maisch) using a mixture of water (solvent A) and 80% acetonitrile in water (solvent B), both acidified with of 0.1% formic acid. Samples were separated at a flow rate of 250 nl min^−1^ within 85 min (5–30% solvent B).

Data were acquired by data-independent acquisition (DIA; full MS, 350–1,200 *m*/*z*; 120,000 resolution; maximum injection time, 60 ms; 28 MS/MS scans with a width of 30.4 *m*/*z*; 1-Da overlap). A normalized automatic gain control target value of 300%, resolution of 30,000 and normalized stepped collision energy of 25.5%, 27% and 30% were used. The MS raw files were processed with Spectronaut 17, direct DIA+, using a full-length *Homo sapiens* database (UniProt, January 2022) and common contaminants, such as trypsin and keratins, as reference.

#### Quantification of proteomics data and statistical analyses

Data analysis was performed using Perseus 2.0.9.0. Values below 5 after log_2_ transformation (result of matching across runs in Spectronaut 17) were transformed to nonvalid values. To determine significant differences in protein abundance, each condition was first compared to the control by standard *t*-test. Only proteins that were significantly more or less abundant in minimal one condition were further analyzed. Missing values were replaced by normally distributed random values. Width and down shift were used separately for each column according to default settings. After grouping replicates, significant changes were determined using a permutation-based false discovery rate (FDR ≤ 0.05).

### Gene set enrichment analysis for transcriptomic and proteomic data

Hallmarks of Cancer and Wikipathways were downloaded from the Molecular Signature Database (MSigDB) website (https://www.gsea-msigdb.org/gsea/msigdb/).

GSEApy (https://gseapy.readthedocs.io/en/latest/introduction.html) was used to quantify the enrichment of pathways in transcriptomic and proteomic data, using a ranked gene list based on log_2_FC as input, with a minimum gene set size of 5, maximum size of 1,000 and 500 permutations. Pathways that were significant at FDR *P* < 0.1 were reported.

### Statistics and reproducibility

No statistical method was used to predetermine sample size. Sample sizes were determined on the basis of previous studies by us^16^ and others^5^ using similar technologies and approaches. For mouse experiments, the number was chosen to comply with 3R principles. No animals or data points were excluded from the analyses. Randomization was used for animal experiments and mice were blindly selected before injection.

All statistical data are based on biological replicates.

Statistical analysis was performed with PRISM software, version 9 for Mac OS X or Windows (GraphPad Software).

All experiments were performed at least twice with similar results.

An unpaired two-sided Student’s *t*-test was used for the comparison of two groups, assuming a normal distribution, which was, however, not formally tested. A Mann–Whitney *U*-test was used for data that were not normally distributed. For comparisons involving more than two groups, we used a two-sided one-way or two-way analysis of variance (ANOVA) with Bonferroni post hoc multiple comparison test.

### Reporting summary

Further information on research design is available in the Nature Portfolio Reporting Summary linked to this article.

## Supplementary information

Reporting Summary

Supplementary Table 1 Supplementary Tables 1–3.

Supplementary Video 1 Real-time holotomographic imaging showing TNTs between cancer cells and fibroblasts.

## Source data

Source Data Fig. 1 Statistical source data.

Source Data Fig. 2 Statistical source data.

Source Data Fig. 3 Statistical source data.

Source Data Fig. 4 Statistical source data.

Source Data Fig. 5 Statistical source data.

Source Data Fig. 6 Statistical source data.

Source Data Fig. 7 Statistical source data.

Source Data Fig. 8 Statistical source data.

Source Data Extended Data Fig. 1 Statistical source data.

Source Data Extended Data Fig. 2 Statistical source data.

Source Data Extended Data Fig. 3 Statistical source data.

Source Data Extended Data Fig. 4 Statistical source data.

Source Data Extended Data Fig. 5 Statistical source data.

Source Data Extended Data Fig. 6 Statistical source data.

Source Data Extended Data Fig. 7 Statistical source data.

Source Data Extended Data Fig. 9 Statistical source data.

Source Data Figs. 1–8 and Extended Data Figs. 1–9 Unprocessed western blots for all figures.

## Data Availability

All data are shown in the article or Supplementary Information. RNA-seq data that support the findings of this study were deposited to the Gene Expression Omnibus under accession number GSE267826. Proteomics data that support the findings of this study are available through ProteomeXchange^71^ with identifier PXD050481. Hallmarks of Cancer and Wikipathways were downloaded from the MSigDB website (https://www.gsea-msigdb.org/gsea/msigdb/). TCGA clinical, survival and RNA-seq data from primary tumors of 8,911 participants across 30 solid cancer types were downloaded from the UCSC Xena data hub (https://xena.ucsc.edu)^61^ using the UCSCXenaTools R package (version 1.4.8)^62^. Source data are provided with this paper.
