# Supplementary material for: MIRO2-mediated mitochondrial transfer from cancer cells induces cancer-associated fibroblast differentiation
Source: Nat Cancer. 2025 Aug 28;6(10):1714–33. doi: 10.1038/s43018-025-01038-6 (PMC12559006; doi:10.1038/s43018-025-01038-6)
Supplement: Supplementary file 1 — Reporting Summary [file 43018_2025_1038_MOESM1_ESM.pdf]

nature portfolio

Corresponding author(s): Michael Cangkrama and Sabine Werner

Last updated by author(s): 17.5.2025

Reporting Summary

Nature Portfolio wishes to improve the reproducibility of the work that we publish. This form provides structure for consistency and transparency in reporting. For further information on Nature Portfolio policies, see our Editorial Policies and the Editorial Policy Checklist.

Statistics

For all statistical analyses, confirm that the following items are present in the figure legend, table legend, main text, or Methods section.

n/a	Confirmed	
☐	☒	The exact sample size (*n*) for each experimental group/condition, given as a discrete number and unit of measurement
☐	☒	A statement on whether measurements were taken from distinct samples or whether the same sample was measured repeatedly
☐	☒	The statistical test(s) used AND whether they are one- or two-sided<br>*Only common tests should be described solely by name; describe more complex techniques in the Methods section.*
☐	☒	A description of all covariates tested
☐	☒	A description of any assumptions or corrections, such as tests of normality and adjustment for multiple comparisons
☐	☒	A full description of the statistical parameters including central tendency (e.g. means) or other basic estimates (e.g. regression coefficient) AND variation (e.g. standard deviation) or associated estimates of uncertainty (e.g. confidence intervals)
☐	☒	For null hypothesis testing, the test statistic (e.g. F, t, r) with confidence intervals, effect sizes, degrees of freedom and P value noted<br>*Give P values as exact values whenever suitable.*
☒	☐	For Bayesian analysis, information on the choice of priors and Markov chain Monte Carlo settings
☒	☐	For hierarchical and complex designs, identification of the appropriate level for tests and full reporting of outcomes
☐	☒	Estimates of effect sizes (e.g. Cohen's *d*, Pearson's *r*), indicating how they were calculated

Our web collection on statistics for biologists contains articles on many of the points above.

Software and code

Policy information about availability of computer code

Data collection

Microscopy and Image Analysis:
Histological analyses and immunostainings were captured using an Axio Imager M2 microscope equipped with an Axiocam MR camera, or an Axioscan 7 slide scanner equipped with a color Axiocam 705 CMOS and fluorescence Axiocam 712 mono cameras (Carl Zeiss AG, Oberkochen, Germany). Image processing and quantification were performed using ZEN 2 software (Carl Zeiss AG) and Fiji/ImageJ (National Institutes of Health). Holotomographic imaging was conducted with a Tomocube HT-X1 microscope (Tomocube Inc.), and images were processed using Tomocube software for real-time 3D visualization.

Proteomics Analysis:
Protein samples were processed for mass spectrometry using SP3 technology. LC-MS/MS was performed on an Orbitrap Exploris 480 mass spectrometer (Thermo Fisher Scientific), and peptides were separated using a Vanquish NEO UHPLC system. Data-independent acquisition (DIA) analysis was conducted with Spectronaut software, using a Homo sapiens UniProt database for protein identification.

Holotomographic Real-Time Imaging:
Real-time cellular imaging was conducted using a Tomocube HT-X1 microscope, with time-lapse videos acquired over 6 h at 5-min intervals. Image processing was performed using Tomocube software for refractive index and fluorescence labeling analysis.

Data analysis

Statistical analysis was performed with PRISM software, version 9 for Mac OS X or Windows (GraphPad Software Inc.).

Fluorescence intensities and cellular spheroid growth were quantified using Fiji/ImageJ software (National Institutes of Health, Bethesda, MD).

Mitochondrial networks were analyzed using the MiNA tool, and mitochondrial distribution was quantified by measuring MitoTracker™ fluorescence intensity using the Fiji plot-profile tool and adapted methods from Delaunay et al. (2022) (Ref. 60).

The length of tunnelling nanotubes between cancer cells and fibroblasts was measured using the line or Polygon tool in QuPath (https://qupath.github.io).

Flow cytometry data were analyzed using FlowJo version 10.10 software (BD Biosciences) (https://www.flowjo.com).

The Cancer Genome Atlas (TCGA) clinical, survival, and RNA-seq data from primary tumors of 8'911 patients across 30 solid cancer types were downloaded from the UCSC Xena data hub (https://xena.ucsc.edu) (Ref. 61) using the 'UCSCXenaTools' R package (version 1.4.8) (Ref 62). Gene expression values were downloaded as log2 transformed RSEM63 normalized counts. The continuous MIRO2 gene expression was used for survival analysis censored at 5 years of follow-up. Hazard ratios were computed using the Cox proportional hazard model implemented in the 'coxph' function from the R package 'survival' (version 3.5-7). For visualization, gene expression was divided into terciles, and Kaplan-Meier survival curves were computed using the R package 'ggsurvfit' (version 0.3.1).

The leading edge (LE) expression signature was composed by 91 genes upregulated at the LE compared to the Tumor Core43. For each TCGA sample, a signature enrichment score was computed using the 'gsva' method from the Gene Set Variation Analysis ('GSVA')64 R/Bioconductor package (version 1.46.0). The correlation between the LE signature score and MIRO2 expression was assessed using Pearson's correlation. Random effects meta-analysis across all cancer types was conducted with the 'metagen' function from the R package 'meta' (version 7.0-0).

Proteomic data were quantified using Perseus 2.0.9.0 software. Values below 5 after Log2 transformation (result of matching across runs in Spectronaut 17) were transformed to non-valid values. To determine significant differences in protein abundance, each condition was first compared to the control by standard t-test.

For quantification of transcriptomics data, FastQ files were trimmed using Trimmomatic (Ref 65) (v0.36), and processed using Salmon (Ref 66) (v1.10.2) using default parameters. The count matrix was processed using tximport (Ref 67) to obtain gene-level counts and transcript per million (TPM) estimates. Principal component analysis was computed on the standardized TPM expression. Differential expression between control, MitoTracker-high, and MitoTracker-low fibroblasts was computed using PyDESeq2 (https://github.com/owkin/PyDESeq2), a Python variant of DESeq2 (Ref 68). Significance levels were cut at 10e-300. To compare the markers of MitoTracker-high fibroblasts with known CAF subtypes, we defined a gene signature for MitoTracker-high fibroblasts using genes that were significantly differentially expressed compared to both MitoTracker-low fibroblasts (adjusted p-value $p<0.01$) and control populations (adjusted p-value $p<0.01$) and over-expressed in the MitoTracker-high population (FC >1.25). We computed the enrichment of this signature in known CAF subtype signatures with Fisher exact test, using genes quantified in the RNA-seq experiment as background.

For spatial deconvolution, Cell2Location was applied to Visium 10X data to estimate cell-type-specific expression profiles. Fibroblast-specific gene expression was mapped to spatial transcriptomic spots, and MIRO2 expression was analyzed in the context of specific CAF subtypes.

Western blot quantification was performed using ImageJ software, normalizing band intensities to loading controls.

"Hallmarks of Cancer" and "Wikipathways" were downloaded from the Molecular Signature Database (MSigDB) website (https://www.gsea-msigdb.org/gsea/msigdb/).

GSEApy (https://gseapy.readthedocs.io/en/latest/introduction.html) was utilized to quantify the enrichment of pathways in transcriptomic and proteomic data, using a ranked gene list based on log2FC as input, with a minimum gene set size of 5, maximum size of 1,000, and 500 permutations. Pathways that were significant by FDR $p<0.1$ were reported.

Species-specific and cell type-specific SNPs were determined by comparing individual chromatograms using SnapGene software (GSL Biotech LLC, San Diego, CA).

The effect of MIRO2 knock-down in genome-wide knock-down screens was analyzed using DepMap (DepMap.org).

For manuscripts utilizing custom algorithms or software that are central to the research but not yet described in published literature, software must be made available to editors and reviewers. We strongly encourage code deposition in a community repository (e.g. GitHub). See the Nature Portfolio guidelines for submitting code & software for further information.

Data

Policy information about availability of data

All manuscripts must include a data availability statement. This statement should provide the following information, where applicable:

- Accession codes, unique identifiers, or web links for publicly available datasets
- A description of any restrictions on data availability
- For clinical datasets or third party data, please ensure that the statement adheres to our policy

All data are shown in the main figures or the Extended Data or the Supplementary Video.
Source data for the main figures and Extended Data figures as well as uncropped Western blots are provided as Source Data files.
RNA-seq data that support the findings of this study have been deposited at the European Nucleotide Archive (ENA), accession number GSE267826 https://www.ncbi.nlm.nih.gov/geo/query/acc.cgi?acc=GSE267826).
Proteomics data that support the findings of this study are available via ProteomeXchange with identifier PXD050481: Project accession: PXD050481.
"Hallmarks of Cancer" and "Wikipathways" were downloaded from the Molecular Signature Database (MSigDB) website (https://www.gsea-msigdb.org/gsea/msigdb/).
The Cancer Genome Atlas (TCGA) clinical, survival, and RNA-seq data from primary tumors of 8'911 patients across 30 solid cancer types were downloaded from the UCSC Xena data hub (https://xena.ucsc.edu) using the 'UCSCXenaTools' R package (version 1.4.8).

Research involving human participants, their data, or biological material

Policy information about studies with human participants or human data. See also policy information about sex, gender (identity/presentation), and sexual orientation and race, ethnicity and racism.

Reporting on sex and gender	Human primary fibroblasts were obtained from foreskin of healthy boys. For the tumor and normal skin samples, which were used for the isolation of primary keratinocytes, we do not have the information if they were from male or female individuals.
Reporting on race, ethnicity, or other socially relevant groupings	All skin samples, which were used for the isolation of primary cells, were obtained from caucasians.
Population characteristics	Foreskin was obtained from healthy boys. Tumor and adjacent skin samples were obtained from adult patients with primary skin SCC (diagnosed by an experienced histopathologist).
Recruitment	All patients that fulfilled the above-described criteria were asked by the surgeon if they provide skin samples.
Ethics oversight	Human skin and tumor samples, which were used for the isolation of primary cells, were obtained anonymously from the Department of Dermatology, University Hospital of Zurich (in the context of the biobank project). Informed consent for use in research was obtained from all donors (in case of foreskin from the parents).<br>All experiments with human samples were approved by the local and cantonal Research Ethics Committees (Kantonale Ethikkommission Zurich, BASEC No. 2017-00684), adhering to the Declaration of Helsinki Principles.

Note that full information on the approval of the study protocol must also be provided in the manuscript.

Field-specific reporting

Please select the one below that is the best fit for your research. If you are not sure, read the appropriate sections before making your selection.

☒ Life sciences ☐ Behavioural & social sciences ☐ Ecological, evolutionary & environmental sciences

For a reference copy of the document with all sections, see nature.com/documents/nr-reporting-summary-flat.pdf

Life sciences study design

All studies must disclose on these points even when the disclosure is negative.

Sample size	No statistical method was used to predetermine sample size. Sample sizes were determined based on previous experience from us (e.g. Ref. 18) and others (e.g. Ref. 71) using similar technologies and approaches. For mouse experiments, the sample sizes were chosen to comply with 3R principles.
Data exclusions	No data or animals were excluded.
Replication	All experiments were performed at least twice with similar results. The exact numbers of biological replicates for each experiment is provided in the figure legends.
Randomization	Allocation was random in the animal experiments.
Blinding	Mice were blindly selected before injection of cancer cells.

Reporting for specific materials, systems and methods

We require information from authors about some types of materials, experimental systems and methods used in many studies. Here, indicate whether each material, system or method listed is relevant to your study. If you are not sure if a list item applies to your research, read the appropriate section before selecting a response.

Materials & experimental systems

n/a	Involved in the study
☐	☒ Antibodies
☐	☒ Eukaryotic cell lines
☒	☐ Palaeontology and archaeology
☐	☒ Animals and other organisms
☒	☐ Clinical data
☒	☐ Dual use research of concern
☒	☐ Plants

Methods

n/a	Involved in the study
☒	☐ ChIP-seq
☐	☒ Flow cytometry
☒	☐ MRI-based neuroimaging

Antibodies

Antibodies used

The following antibodies were used for Western blot analysis:
anti-MIRO2 (#H00089941-B01P, Novus Biologicals, 1:1,000 diluted), mouse polyclonal antibody
anti-MIRO1 (NBP1-59021, Novus Biologicals; 1:500 diluted), rabbit polyclonal antibody
anti-TRAK1 (#PA5-70029, Invitrogen/Thermo Fisher Scientific, 1:500 diluted), rabbit polyclonal antibody
anti-TRAK2 (#PA5-21858, Invitrogen, 1:500 diluted), mouse polyclonal antibody
anti-EXOC1 (ab251853, Abcam, 1:500 diluted), rabbit polyclonal antibody
anti-EXOC2 (ab14062, Abcam, 1:500 diluted), rabbit monoclonal antibody
anti-vinculin (V4505, Sigma-Aldrich, 1:2,000 diluted), mouse monoclonal antibody
anti-GAPDH ((#5G4; Hytest;1:10,000 diluted), mouse monoclonal antibody
anti-HSP60 (Ab59457, Abcam, 1:500 diluted), mouse monoclonal antibody
anti-rabbit IgG (W4011, Promega; 1:10,000 diluted) conjugated with horseradish peroxidase, goat polyclonal antibody
anti-mouse IgG (W4021, Promegal, 1:10,000 diluted), conjugated with horseradish peroxidase, goat polyclonal antibody

The following antibodies were used for immunofluorescence staining:

Alexa Fluor® 488 AffiniPuredonkey anti-mouse IgG (H+L) (Jackson ImmunoResearch, #715-545-150; 1:200 diluted), donkey polyclonal antibody
Alexa Fluor® 594 AffiniPure donkey anti-mouse IgG (H+L) (Jackson ImmunoResearch, #715-585-150, 1:200 diluted), donkey polyclonal antibody
Alexa Fluor® 488 AffiniPure donkey anti-rabbit IgG (H+L) (Jackson ImmunoResearch, #711-547-003, 1:200 diluted), donkey polyclonal antibody
Alexa Fluor® 594 AffiniPure donkey anti-rabbit IgG (H+L) (Jackson ImmunoResearch, #711-587-003, 1:200 diluted), donkey polyclona antibody
Alexa Fluor® 594 AffiniPure donkey anti-goat IgG (H+L) (Jackson ImmunoResearch, #705-585-147; 1:200 diluted), donkey polyclonal antibody
anti-E-cadherin (Pharmingen, #610181, 1:500 diluted), mouse monoclonal antibody
anti-fibronectin 1 (Abcam, ab2413, 1:500 diluted), rabbit polyclonal antibody
anti-Ki67 (Abcam, ab16667, 1:500 diluted), rabbit monoclonal antibody
anti-vimentin (Abcam, ab92547, 1:500 diluted), rabbit monoclonal antibody
anti-keratin 14 (K14) BioLegend, PRB-155P, 1:500 diluted), rabbit polyclonal antibody
anti-MECA32 (BD Pharmingen, #553849, 1:1000 diluted), rat monoclonal antibody
anti-type I collagen (BioConcept, (#1310-01, 1:500 diluted), goat polyclonal antibody
anti-PDGFRA (Santa Cruz, #sc-431, 1:500 diluted), rabbit polyclonal antibody
anti-MIRO2 (#H00089941-B01P, Novus Biologicals, 1:500 diluted), mouse polyclonal antibody
anti-human mitochondria antibody (Abcam, [113-1]; ab92824, 1:500 diluted, mouse monoclonal antibody

Validation

Validation information for the antibodies listed above:

anti-MIRO2: validated by the manufacturer for detection of human MIRO2 (RHOT2) by Western blot and immunofluorescence staining
anti-MIRO1: validated by the manufacturer for detection of human MIRO1 (RHOT1) by Western blot, immunoprecipitation and immunohistochemistry
anti-TRAK1: validated by the manufacturer for detection of human TRAK1 by Western blot
anti-TRAK2: validated by the manufacturer for detection of human TRAK2 by Western blot and immunocytochemistry
anti-EXOC1: validated by the manufacturer for detection of human EXOC1 by Western blot and immunocytochemistry
anti-EXOC2: validated by the manufacturer for detection of human, mouse and rat EXOC2 by Western blot and flow cytometry
anti-vinculin: validated by the manufacturer for detection of human, mouse, chicken and bovine vinculin by Western blot, microarrray and immunofluorescence staining
anti-GAPDH: validated by the manufacturer for detection of human, bovine, porcine, goat, canine, rabbit, cat, rat, mouse and fish GAPDH by Western blot and immunocytochemistry
anti-HSP60: validated by the manufacturer for detection of human, rat and mouse HSP60 by Western blot, immunocytochemistry, immunohistochemistry, and flow cytometry

anti-rabbit IgG, HRP-conjugated: validated by the manufacturer for detection of rabbit IgG by Western blot, ELISA and dot blotting
anti-mouse IgG, HRP-conjugated: validated by the manufacturer for detection of mouse IgG by Western blot, ELISA and dot blotting

Alexa Fluor® 488 AffiniPuredonkey anti-mouse IgG (H+L): validated by the manufacturer for detection of mouse IgG by immunofluorescence staining
Alexa Fluor® 594 AffiniPure donkey anti-mouse IgG (H+L): validated by the manufacturer for detection of mouse IgG by immunofluorescence staining
Alexa Fluor® 488 AffiniPure donkey anti-rabbit IgG (H+L): validated by the manufacturer for detection of rabbit IgG by immunofluorescence staining
Alexa Fluor® 594 AffiniPure donkey anti-rabbit IgG (H+L): validated by the manufacturer for detection of rabbit IgG by immunofluorescence staining
Alexa Fluor® 594 AffiniPure donkey anti-goat IgG (H+L): validated by the manufacturer for detection of goat IgG by immunofluorescence staining
anti-E-cadherin: validated by the manufacturer for detection of human, mouse, rat and dog E-cadherin by Western blot, immunofluorescence, immunohistochemistry, immunoprecipitation
anti-fibronectin: validated by the manufacturer for detection of human and mouse fibronectin by Western blot, immunohistochemistry and immunfluorescence staining
anti-Ki67: validated by the manufacturer for detection of human, mouse and rat Ki67 by Western blot , immunohistochemistry, immunofluorescence staining, and flow cytometry
anti-vimentin: validated by the manufacturer for detection of human, mouse and rat vimentin by Western blot, immunohistochemistry, immunofluorescence staining and flow cytometry
anti-keratin 14 (K14): validated by the manufacturer for detection of human ad mouse keratin 14 by Western blot, immunocytochemistry and immunohistochemistry
anti-MECA32:validated by the manufacturer for detection of mouse Meca32 by flow cytometry, immunoprecipiation, immunohistochemistry, immunocytochemistry and Western blot
anti-type I collagen: validated by the manufacturer for detection of type I collagen from humans, mice, rats and several other species by Western blot, immunoprecipitation, immunohistochemistry, immunofluorescence staining, flow cytometry and immunoprecipitation
anti-PDGFRA: validated by the manufacturer for detection of PDGFRA from humans, mice, rats and several other species by Western blot, immunohistochemistry, immunofluorescence staining and immunoprecipitation.
anti-MIRO2: validated by the manufacturer for detection of human MIRO2 (RHOT2) by Western blot and immunofluorescence staining
anti-human mitochondria: validated by the manufacturer for detection of human mitochondria by Western blot, immunohistochemistry, immunofluorescence staining and flow cytometry.

Eukaryotic cell lines

Policy information about cell lines and Sex and Gender in Research

Cell line source(s)	HaCaT, HaCaT-Ras cells and SCC13 cells were provided by Dr. Petra Boukamp, Düsseldorf, Germany. A431 cells were from Merck (Darmstadt, Germany; #85090402). LifeACT cell lines were generated by transduction with the lentiviral vector rLV-Ubi-LifeAct RFP-Tag (Vitaris, Baar, Switzerland). HaCaT cells are spontaneously immortalized, but non-tumorigenic human keratinocytes(Ref. 21). HaCaT-Ras cells had been obtained by transfection of HaCaT cells with a c-Ha-ras oncogene (Ref. 32). SCC13 cells were derived from a human cutaneous SCC (Ref 31). The metastatic MDA-MB231 triple negative breast cancer cell line and the metastatic PANC1 pancreatic cancer cell line were obtained from ATCC (Manassas, VA, #HTB-26, #CRL-1469). LM2 cells, a lung metastatic variant of MDA-MB-231, were kindly provided by Dr. Joan Massagué, New York, NY. MDA231. This variant was established after 3 passages in nude mice. Cell lines expressing fluorescent mitochondrial proteins were generated via lentiviral transduction (Ref. 35, 55). Primary fibroblasts expressing TOM20-GFP were generated by lentiviral transduction with pLenti-X1-blast-GFP-TOM20-MTS, provided by Dr. Jacob Corn, ETH Zurich, Switzerland. Immortalized mouse fibroblasts had been isolated from PDGFRα-eGFP transgenic mice and spontaneously immortalized via serial passaging (Ref. 56).
Authentication	Authentication of HaCaT, HaCaT-Ras, SCC13 and A431 cells was performed by Microsynth AG, Balgach, Switzerland, using highly polymorphic short tandem repeat loci (STRs); most recently in February 2025. Breast and pancreatic cancer cell lines were not authenticated by the investigators.
Mycoplasma contamination	All cell lines and primary cells were tested negative for mycoplasma contamination.
Commonly misidentified lines (See ICLAC register)	No commonly misidentified cells lines were used.

Animals and other research organisms

Policy information about studies involving animals; ARRIVE guidelines recommended for reporting animal research, and Sex and Gender in Research

Laboratory animals	NOD/SCID (NOD.CB17-Prkdcscid/NCrCrl) were used for tumor xenograft experiments at the age of 10-14 weeks.
Wild animals	No wild animals were used.
Reporting on sex	Male mice were used for the skin cancer xenografts, and female mice were used for the breast cancer xenografts.

Field-collected samples | The study did not involve samples collected in the field.

Ethics oversight | Mouse maintenance and experimentation had been approved by the veterinary authorities of Zurich, Switzerland (Kantonales Veterinäramt Zurich, #32060, #35555 #36338 and #33866).
The maximal tumor size permitted by the ethics committee (1 cm diameter for skin cancer, 2.8 cm2 volume for breast cancer) or the end point for wellbeing (pancreatic cancer) was never reached in our experiments.

Note that full information on the approval of the study protocol must also be provided in the manuscript.

Plants

Seed stocks | This study did not involve plants

Novel plant genotypes | This study did not involve plants

Authentication | This study did not involve plants

Flow Cytometry

Plots

Confirm that:

☒ The axis labels state the marker and fluorochrome used (e.g. CD4-FITC).

☒ The axis scales are clearly visible. Include numbers along axes only for bottom left plot of group (a 'group' is an analysis of identical markers).

☒ All plots are contour plots with outliers or pseudocolor plots.

☒ A numerical value for number of cells or percentage (with statistics) is provided.

Methodology

Sample preparation | To prepare the cell samples for flow cytometry analysis, cells were first trypsinized to facilitate detachment from culture dishes. Following trypsinization, the cells were neutralized and collected by centrifugation. The cell pellet was then resuspended in phosphate-buffered saline (PBS) containing 1% fetal bovine serum (FBS) to provide a suitable environment for the cells during analysis. This suspension ensures that the cells remain viable and in a single-cell state, which is essential for accurate flow cytometry readings. The prepared samples were then passed through a flow cytometer for analysis.

Instrument | Co-cultured cells were sorted according to their distinct fluorescence profiles using a BD FACSAriaTM Fusion sorter (Becton Dickinson, Franklin Lakes, NJ)

Software | FlowJo version 10.10

Cell population abundance | Experiments did not involve post-sort fractions

Gating strategy | Gating strategy used to identify high or low MitoTracker cell population subsets is shown in ED Figure 2a.Gating strategy for Su9-GFP positive cells is shown in ED Figure 3e.

☒ Tick this box to confirm that a figure exemplifying the gating strategy is provided in the Supplementary Information.
