# Supplementary material for: MIRO2-mediated mitochondrial transfer from cancer cells induces cancer-associated fibroblast differentiation
Source: Nat Cancer. 2025 Aug 28;6(10):1714–33. doi: 10.1038/s43018-025-01038-6 (PMC12559006; doi:10.1038/s43018-025-01038-6)
Supplement: Supplementary file 20 — Unprocessed western blots for all figures. [file 43018_2025_1038_MOESM20_ESM.pdf]

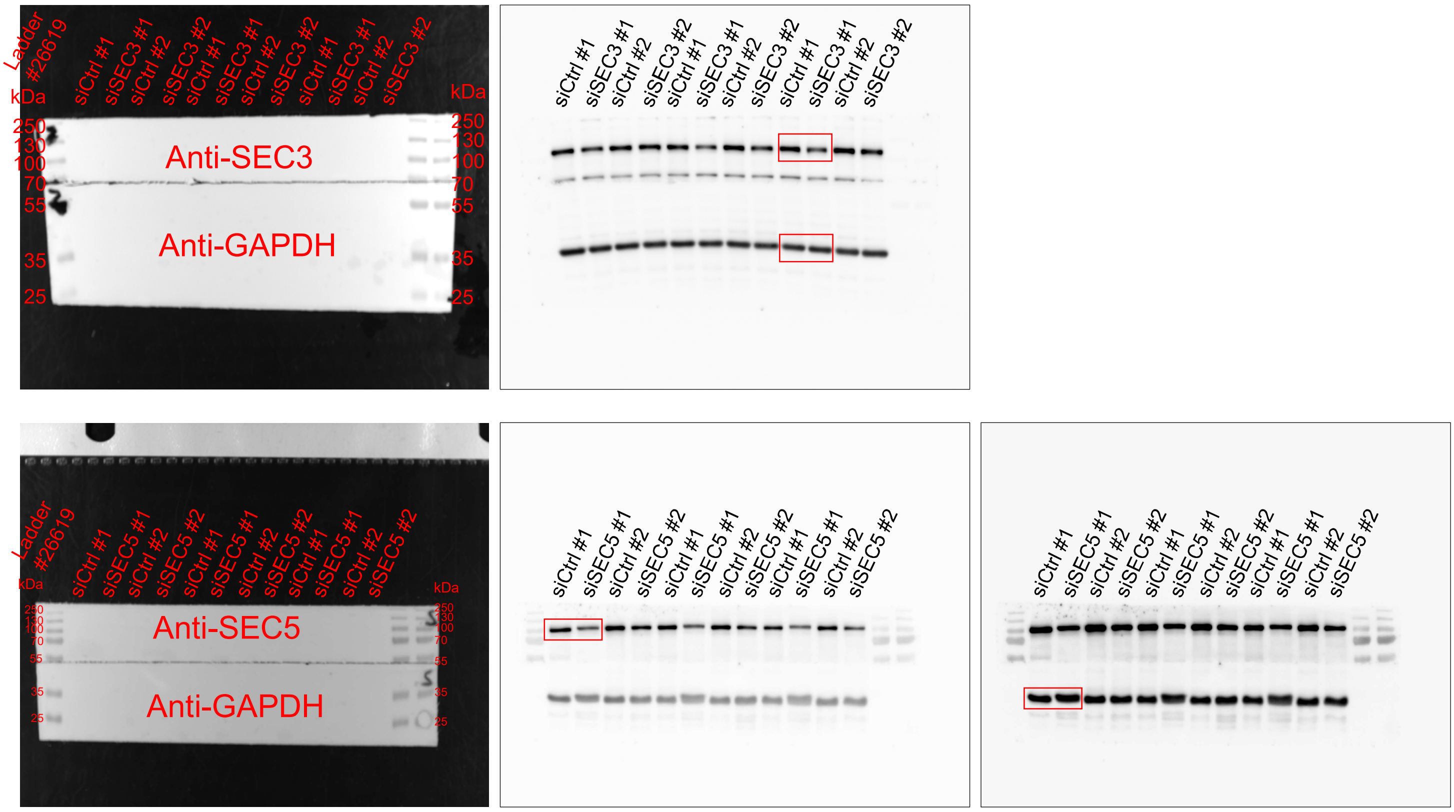

Fig. 1j: Western blot for SEC3 (EXOC1) and SEC5 (EXOC2) using A431 cells transfected with siCtrl, siSEC3 or siSEC5.

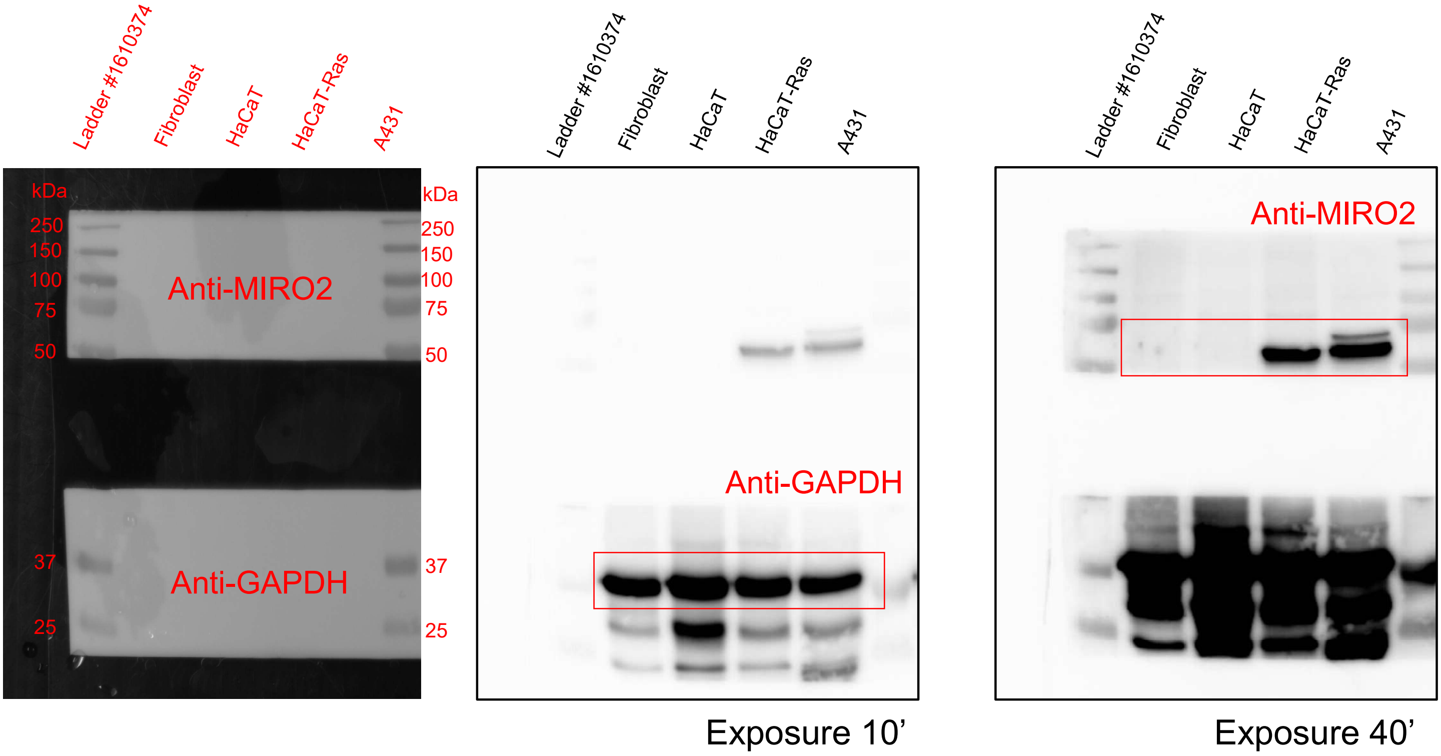

Fig. 6d: Western blot of total lysates from HPF, HaCaT, HaCaT-Ras and A431 cells for MIRO2 and GAPDH.

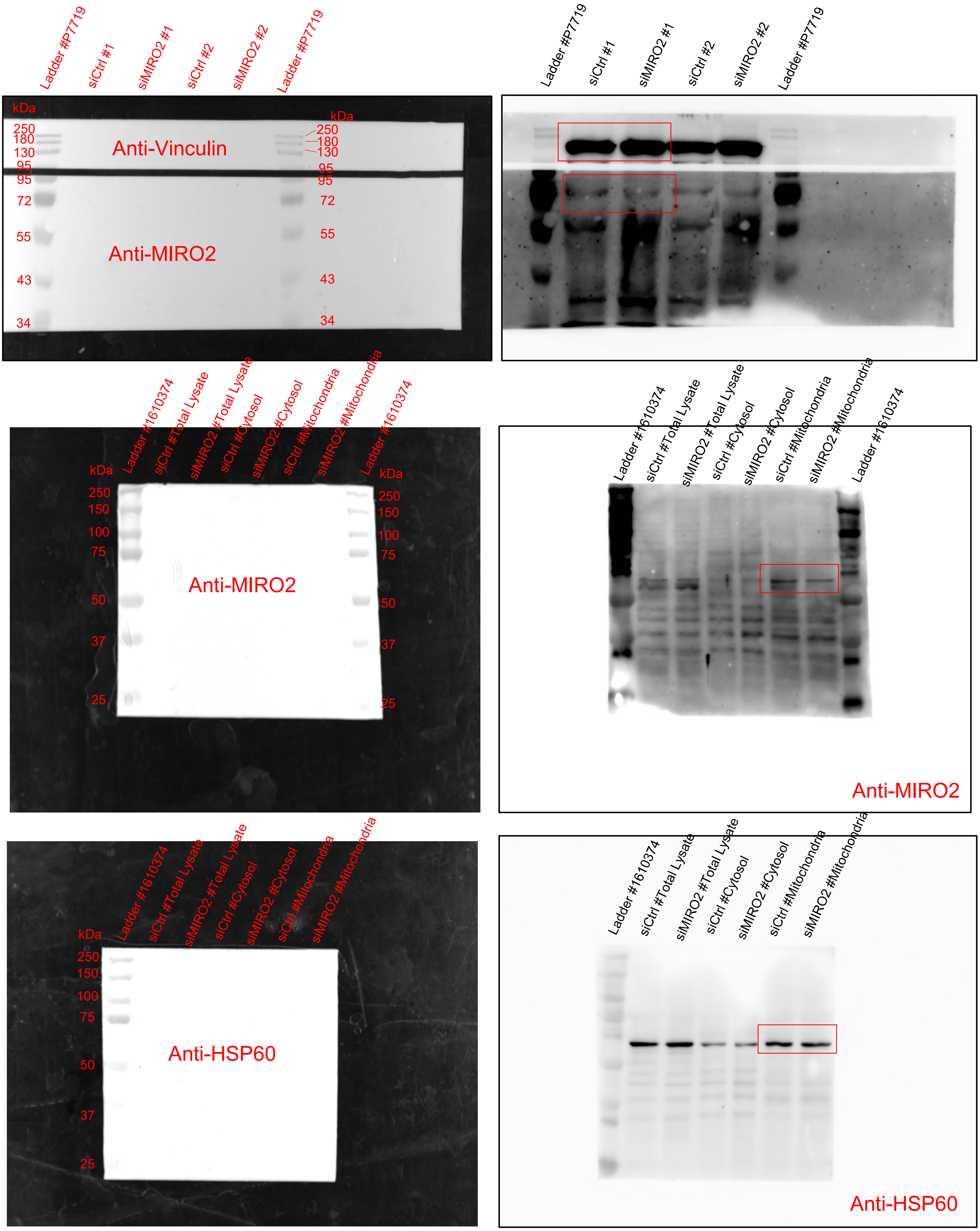

Fig. 7a: Western blot of total and mitochondrial lysates from siCtrl or siMIRO2 A431 cells for MIRO2, vinculin or HSP60 (loading controls)

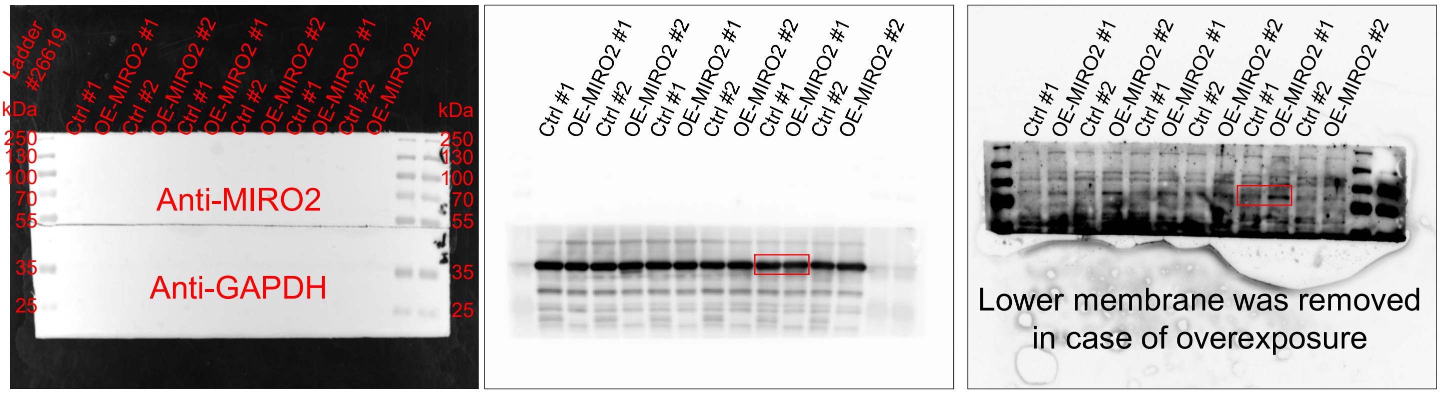

Fig. 7j: Western blot of total lysates from control or MIRO2-overexpressing A431 cells for MIRO2, or GAPDH (loading controls).

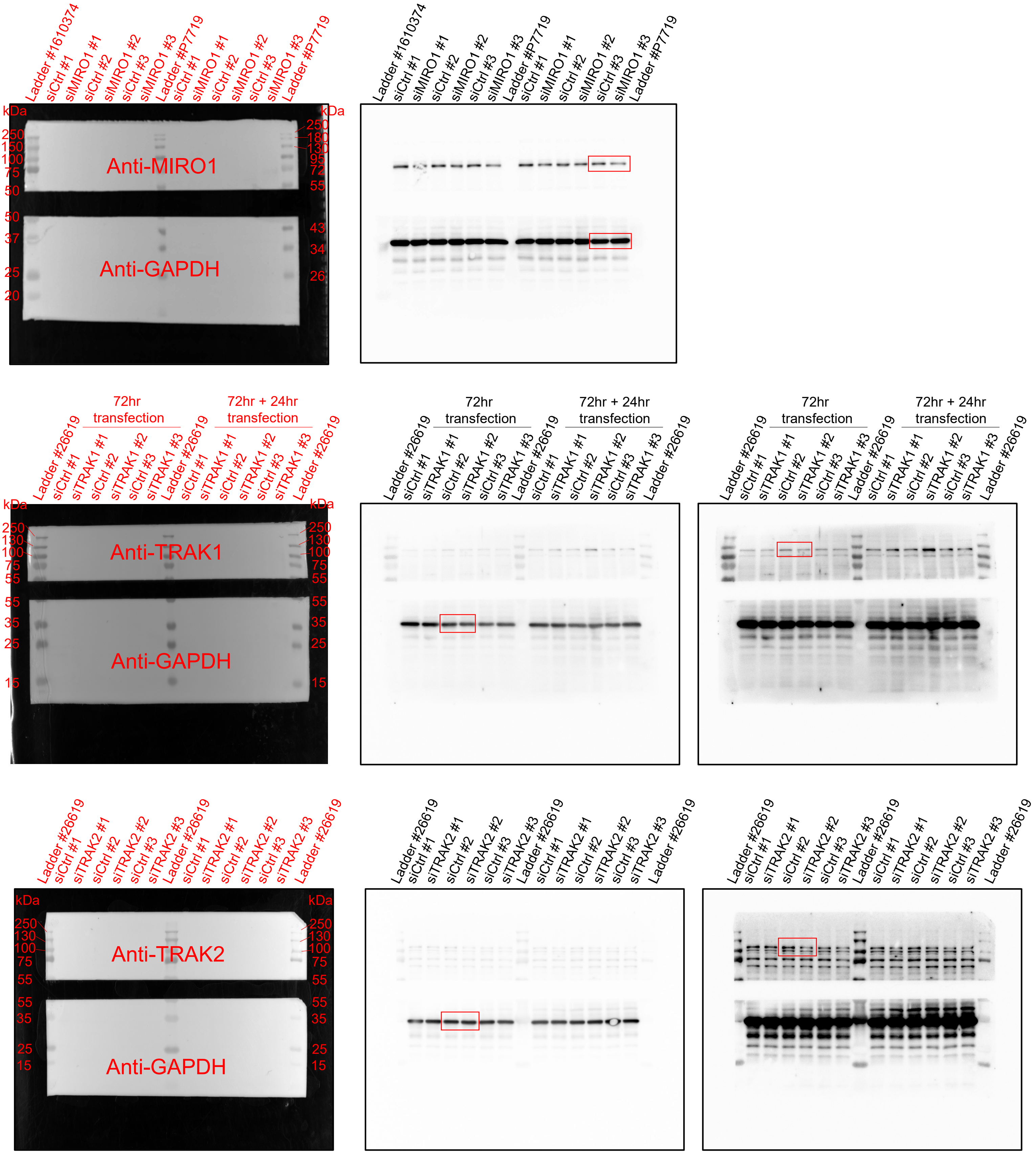

Extended Data Fig. 9c: Western blot analysis of total lysates from A431 cells transfected with siCtrl, siMIRO1, siTRAK1, or siTRAK2 for MIRO1, TRAK1, TRAK2, and GAPDH (loading control).

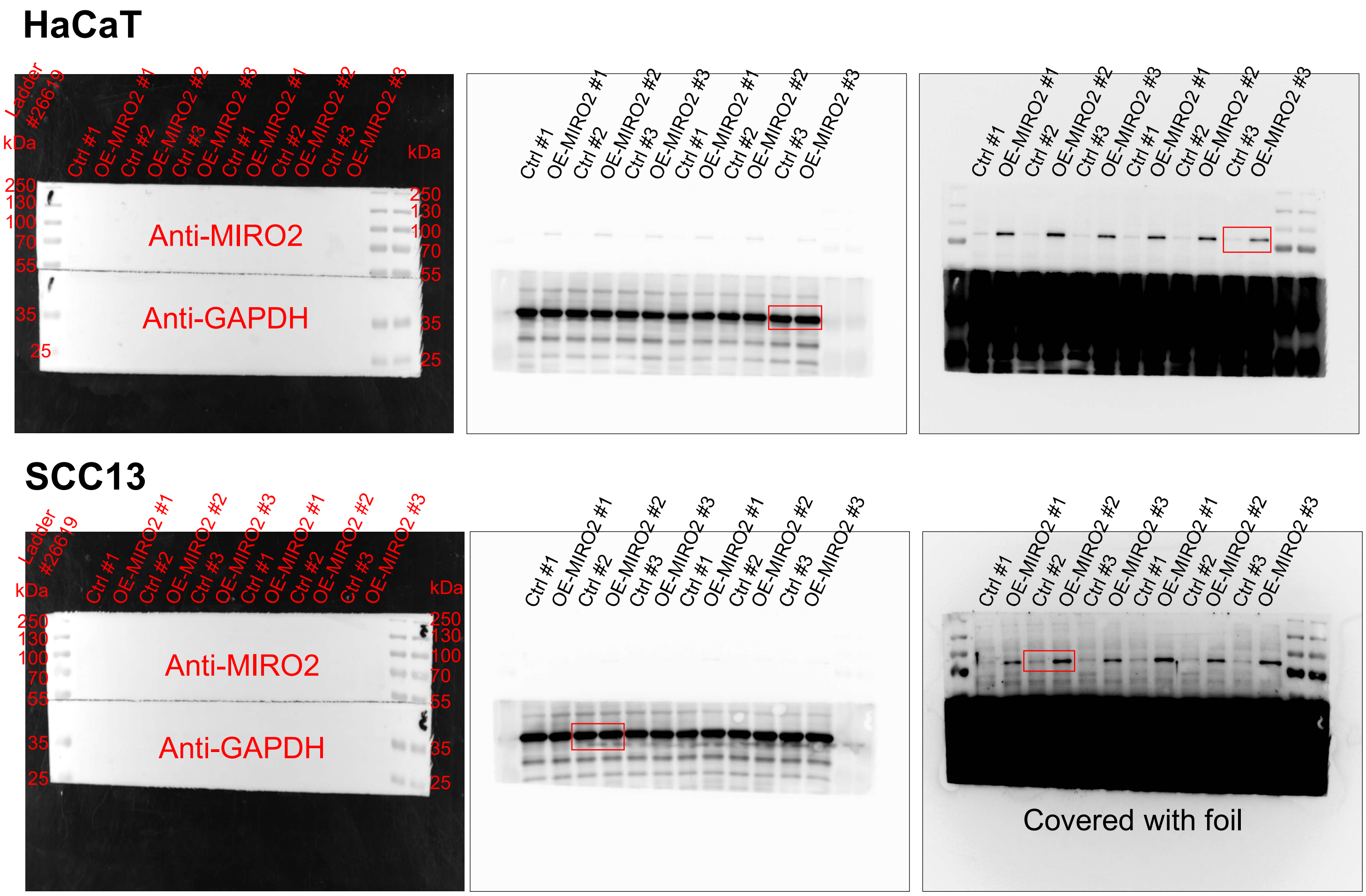

Extended Data Fig. 9f: Western blot for MIRO2 using total lysates from HaCaT or SCC13 cells transfected with a control or a MIRO2 overexpression vector (OE-MIRO2).
